# Human genome-edited hematopoietic stem cells phenotypically correct Mucopolysaccharidosis type I

**DOI:** 10.1038/s41467-019-11962-8

**Published:** 2019-09-06

**Authors:** Natalia Gomez-Ospina, Samantha G. Scharenberg, Nathalie Mostrel, Rasmus O. Bak, Sruthi Mantri, Rolen M. Quadros, Channabasavaiah B. Gurumurthy, Ciaran Lee, Gang Bao, Carlos J. Suarez, Shaukat Khan, Kazuki Sawamoto, Shunji Tomatsu, Nitin Raj, Laura D. Attardi, Laure Aurelian, Matthew H. Porteus

**Affiliations:** 1 Department of Pediatrics, Stanford University School of Medicine, Stanford, CA USA; 2 Department of Biomedicine, Aarhus University, DK-8000 Aarhus C., Denmark; 3 Aarhus Institute of Advanced Studies (AIAS), Aarhus University, DK-8000 Aarhus C., Denmark; 4 Mouse Genome Engineering Core Facility, Vice Chancellor for Research Office, University of Nebraska Medical Center, Omaha, NE USA; 5 Department of Pharmacology and Experimental Neuroscience, College of Medicine, University of Nebraska Medical Center, Omaha, NE USA; 6 Department of Bioengineering, Rice University, Houston, TX USA; 7 Department of Pathology, Stanford University School of Medicine, Stanford, CA USA; 8 Nemours/ Alfred I. duPont Hospital for Children, Wilmington, DE 19803 USA; 9 Department of Radiation Oncology, Stanford University School of Medicine, Stanford, CA 94305 USA; 10 Department of Genetics, Stanford University School of Medicine, Stanford, CA 94305 USA; 11 Stanford University School of Medicine, Stanford, CA 94305 USA; 12 University of Maryland School of Medicine, Baltimore, MD 21201 USA

**Keywords:** Targeted gene repair, CRISPR-Cas9 genome editing

## Abstract

Lysosomal enzyme deficiencies comprise a large group of genetic disorders that generally lack effective treatments. A potential treatment approach is to engineer the patient’s own hematopoietic system to express high levels of the deficient enzyme, thereby correcting the biochemical defect and halting disease progression. Here, we present an efficient ex vivo genome editing approach using CRISPR-Cas9 that targets the lysosomal enzyme iduronidase to the *CCR5* safe harbor locus in human CD34+ hematopoietic stem and progenitor cells. The modified cells secrete supra-endogenous enzyme levels, maintain long-term repopulation and multi-lineage differentiation potential, and can improve biochemical and phenotypic abnormalities in an immunocompromised mouse model of Mucopolysaccharidosis type I. These studies provide support for the development of genome-edited CD34+ hematopoietic stem and progenitor cells as a potential treatment for Mucopolysaccharidosis type I. The safe harbor approach constitutes a flexible platform for the expression of lysosomal enzymes making it applicable to other lysosomal storage disorders.

## Abstract (web summary)

Mucopolysaccharidosis type I (MPSI) is a lysosomal storage disease caused by insufficient iduronidase (IDUA) activity. Here, the authors use an ex vivo genome editing approach to overexpress IDUA in human hematopoietic stem and progenitor cells and show it can phenotypically correct MSPI in mouse model.

## Introduction

Lysosomal storage diseases (LSDs) comprise a large group of genetic disorders caused by deficiencies in lysosomal proteins; many lack effective treatments. Mucopolysaccharidosis type I (MPSI) is a common LSD caused by insufficient iduronidase (IDUA) activity that results in glycosaminoglycan (GAG) accumulation and progressive multi-systemic deterioration that severely affects the neurological and musculoskeletal systems^1^. Current interventions for MPSI include enzyme replacement therapy (ERT) and allogeneic hematopoietic stem cell transplantation (allo-HSCT); both have limited efficacy. ERT does not cross the blood–brain barrier, requires costly life-long infusions, and inhibitory antibodies can further decrease enzyme bioavailability^2^. Allo-HSCT results in better outcomes than ERT by providing a persistent source of enzyme and tissue macrophages that can migrate into affected organs, including the brain, to deliver local enzyme^3–5^. However, allo-HSCT has significant limitations, including the uncertain availability of suitable donors, delay in treatment (allowing for irreversible progression), and transplant-associated morbidity and mortality such as graft-versus-host disease and drug-induced immunosuppression.

Human and animal studies in MPSI have shown that the therapeutic efficacy of HSCT can be enhanced by increasing the levels of circulating IDUA. In humans, patients transplanted with non-carrier donors had better clinical responses than patients transplanted with HSPCs from MPSI heterozygotes with decreased enzyme expression^6^. In mice, transplantation of virally transduced murine hematopoietic stem and progenitor cells (HSPCs) expressing supra-normal enzyme levels^7,8^ dramatically corrected the phenotype. Based on this, autologous transplantation of lentivirus-transduced HSPCs overexpressing lysosomal enzymes is being explored in human trials for LSDs including severe MPSI (ClinicalTrials.gov, NCT03488394) and Metachromatic leukodystrophy^9^. The peroxisomal disorder X-linked adrenoleukodystrophy has also been successfully treated by lentiviral transduced autologous HSPCs though supra-normal expression of the missing enzyme is probably not critical as cross-correction is not a feature of this disease^10,11^. This autologous approach eliminates the need to find immunologically matched donors and reduces some of the potential complications from allogeneic transplants. However, concerns remain about the potential for tumorigenicity associated with random insertion of the viral genomes^12,13^, carry-over of infectious particles^14^, the immune response to some of the vectors, and variable transgene expression^15^.

Recently developed genome editing tools combine precise gene addition with genetic alterations that can add therapeutic benefit^16^. Among these, Clustered Regularly Interspaced Short Palindromic Repeats-associated protein-9 nuclease (CRISPR/Cas9) is the simplest to engineer and has been used to successfully modify HSPCs in culture^17,18^. The system was repurposed for editing eukaryotic cells by delivering the Cas9 nuclease, and a short guide RNA (sgRNA). When targeted to the sequence determined by the sgRNA, Cas9 creates a double-stranded DNA break, thereby stimulating homologous recombination with a designed donor DNA template that contains the desired genetic modification embedded between homology arms centered at the break site. This process, termed “homologous recombination-mediated genome editing” (HR-GE) is most often used for in situ gene correction and has been hailed as a tool to treat monogenic diseases. Although its therapeutic potential in LSDs remains to be explored, to maximize therapeutic correction by autologous transplantation of genetically modified HSPCs in some LSDs, functional enzymes must sometimes be expressed at higher-than-endogenous levels. This can be achieved by inserting an expression cassette (exogenous promoter-gene of interest) into non-essential genomic region (or “safe harbor”). A safe harbor provides a platform that is independent of specific patient mutations, is easily adaptable to various lysosomal enzymes and, compared to lentiviral transduction, ensures more predictable and consistent transgene expression because the insertion sites are restricted (up to 2 in autosomes). Moreover, its disruption has no effect on cell proliferation and no known potential for oncogenic transformation.

We describe the development of such an approach for MPSI. We use *CCR5* as the target safe harbor to insert an expression cassette to overexpress IDUA in human CD34+ HPSCs and their progeny. *CCR5* is considered a non-essential gene because bi-allelic inactivation of *CCR5* (CCR5∆32) has no general detrimental impact on human health and the only known phenotypes of CCR5 loss are resistance to HIV-1 infection and increased susceptibility to West Nile virus^19^. We report that human HSPCs modified using genome editing to express IDUA from the *CCR5* locus engraft and ameliorate biochemical, visceral, musculoskeletal, and neurologic manifestations of the disease in a new immunocompromised model of MSPI.

## Results

### Efficient targeting of IDUA to the *CCR5* locus in human HSPCs

To generate human CD34^+^ HPSCs overexpressing IDUA, we used sgRNA/Cas9 ribonucleoprotein (RNP) and adeno-associated viral vector serotype six (AAV6) delivery of the homologous templates^20^. RNP complexes consisting of 2′-*O*-methyl 3′phosphorothioate-modified *CCR5* sgRNA^21^ and Cas9 protein were electroporated into cord blood-derived (CB) and adult peripheral blood-derived HSPCs (PB). The efficiency of double-strand DNA break (DSB) generation by our *CCR5* RNP complex was estimated by measuring the frequency of insertions/deletions (Indel) at the predicted cut site. The mean Indel frequencies were 83% ± 8 (±SD) in CB-HSPCs and 76% ± 8 in PB-HSPCs, consistent with a highly active sgRNA. The predominant Indel was a single A/T insertion that abrogated CCR5 protein expression (Supplementary Fig. 1)^22^.

**Fig. 1:**
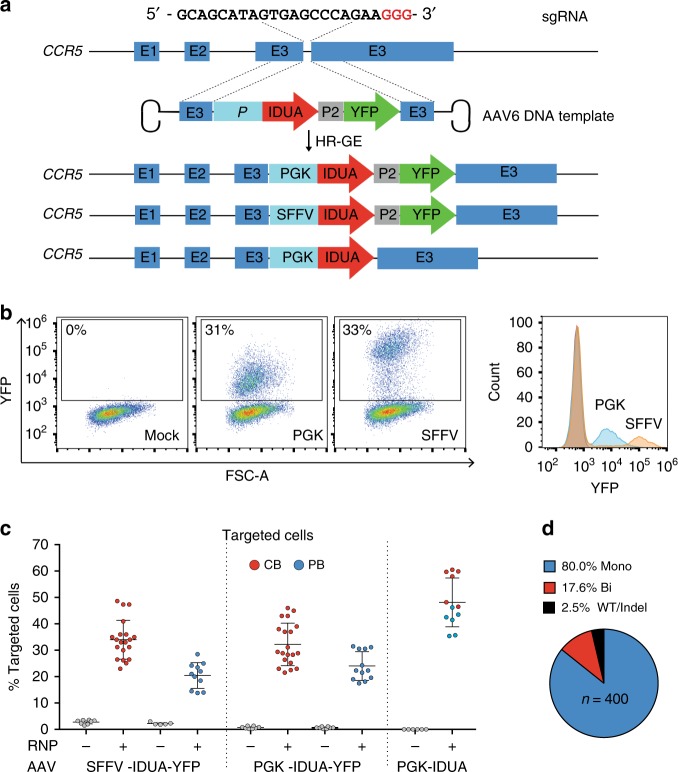
Efficient CRIPR/Cas9-mediated integration of IDUA overexpression cassettes into the *CCR5* locus in human CD34+ HSPCs. **a** Schematic of targeted integration of IDUA and expression cassettes. The AAV6 genome was constructed to have 500 bp arms of homology centered on the cut site, and the IDUA sequence placed under the control of the SFFV or the PGK promoter (E = Exon). In two DNA templates, YFP was expressed downstream of IDUA using the self-cleaving P2A peptide. **b** Representative FACs and histogram plots 3-days post-modification of mock and human HSPCs that underwent RNP and AAV6 exposure with YFP-containing expression cassettes. **c** Targeting frequencies in cord blood (CB, red dots) and adult peripheral blood (PB, blue dots)-derived HSPCs read by percent fluorescent cells in YFP-expressing cassettes and percent colonies with targeted CCR5 alleles by single cell-derived colony genotyping in cassettes without the reporter. Each dot represents the average of duplicates for a human cell donor. For RNP + AAV6 conditions with YFP templates, *n* = 20 and *n* = 11 independent human donors for CB and PB respectively. For the template without selection *n* = 6 independent human donors in CB and PB. Data shown as mean ± SD. **d** Distribution of wild type (WT), mono and bi-allelically modified cells (*n* = 400) in YFP-positive HSPCs

To achieve precise genetic modification, the templates for homologous recombination were made by inserting IDUA expression cassettes driven by the spleen focus-forming virus (SFFV) or the phosphoglycerate kinase (PGK) promoter, followed by a yellow fluorescent protein (YFP) downstream of the self-cleaving P2A peptide into the AAV vector genome. A third expression cassette containing IDUA driven by PGK but without a selection marker was also made (Fig. 1a). These strong constitutive promoters were chosen to harness the ability of all hematopoietic lineages to express IDUA and maximize biochemical cross-correction, and because IDUA expression was previously shown not be toxic to HSPCs. Following electroporation, CB and PB cells transduced with the SFFV-IDUA-YFP and PGK-IDUA-YFP viruses were examined for YFP fluorescence to quantify the efficiency of modification. As shown in Fig. 1b, RNP electroporation followed by AAV6 transduction lead to a marked increase in the median fluorescence intensity of the cells. As previously reported, this shift in the fluorescence intensity allows for identification of cells that have successfully undergone HR-GE^18^. In CB-derived HSPCs the mean fraction of YFP-positive cells, was 34% ± 7 and 32% ± 8 with SFFV and PGK-driven expression cassettes respectively. In PB-HSPCs, the frequencies were 21% ± 5, and 24% ± 5 for the same AAV6 donors (Fig. 1c). AAV6 transduction alone showed <2% YFP-positive cells, while mock cells that underwent electroporation but not AAV transduction had no detectable fluorescence. We measured the efficiency of modification in CB and PB cells transduced with the PGK-IDUA virus lacking the reporter (PGK-IDUA) by genotyping single cell-derived colonies from colony formation assays (CFAs) (Supplementary Fig. 2a, b). In these cells, the frequencies of modification were 54% ± 10, and 44% ± 7 in CB and PB-HSPCs, considerably higher than the larger, YFP-containing cassettes, suggesting that efficiency is dependent on insert size (Fig. 1c). Based on these targeting frequencies we conclude that our genome editing protocol is efficient and reproducible for human CB and PB-derived HSPCs.

We also characterized the genomic modifications at *CCR5* loci, by quantifying the fraction of targeted alleles in bulk DNA preparations using droplet-digital PCR (ddPCR) (Supplementary Fig. 2c, d). This data allowed us to estimate the distribution of cells with one (mono-allelic) or two (bi-allelic) alleles targeted in different cell donor samples and indicated that, for the YFP constructs, 65–100% of the cells had mono-allelic modification (Supplementary Note 1 and Supplementary Table 1). Consistent with this, genotyping of YFP-positive colonies in CFAs showed a mean mono-allelic modification frequency of 80% ± 7.5 (Fig. 1d).

### High IDUA expression in edited HSPCs and derived macrophages

**Fig. 2:**
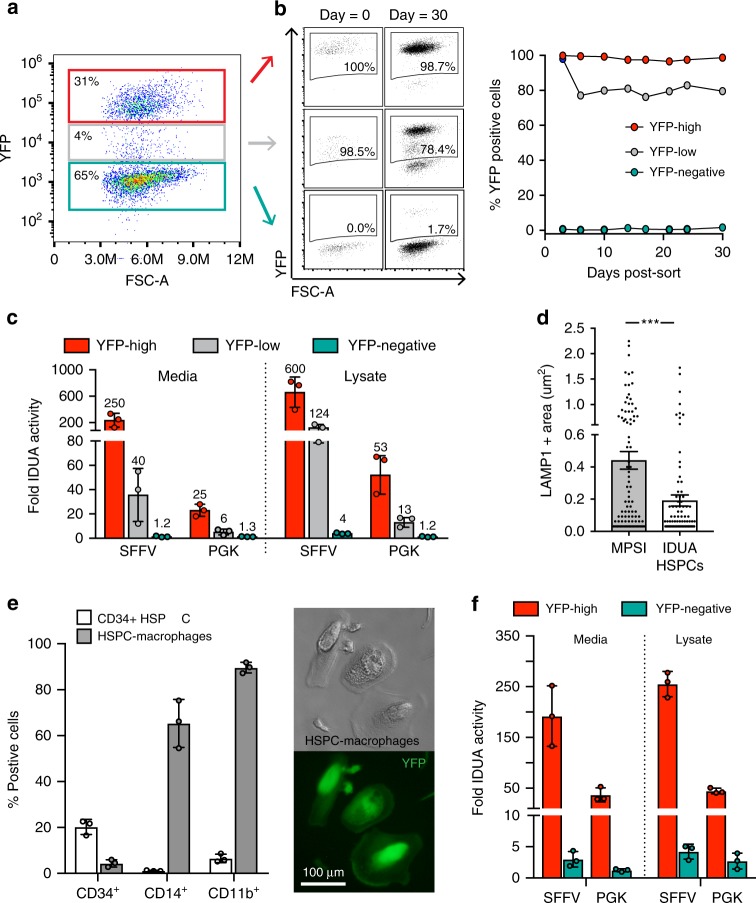
Enhanced IDUA expression by IDUA-HSPCs and derived macrophages. **a** Representative FACS plot showing three distinct populations based on YFP expression 3 days post-modification. **b** Percent YFP-positive cells in culture (30 days). **c**, Fold increase in IDUA secretion and intracellular expression by YFP-high, YFP-low, and YFP-negative populations compared to mock cells. **d**, Average LAMP-1 + area in MPSI fibroblasts co-cultured with IDUA-HSPCs. Each dot represents a cell. **e**, Human CD34, CD14, and CD11b marker expression in HSPC-derived macrophages after in vitro differentiation compared to undifferentiated cells (CD34 + HSPCs). Macrophage morphology and YFP expression after differentiation. **f**, Fold increase in IDUA secretion and intracellular expression in HSPC-macrophages modified with SFFV and PGK expression cassettes. **c, e, f**, Each column represents average of triplicates in *n* = 3 independent biological samples. All data expressed as mean ± SD, ***p < .001 in two-sided unpaired t-test. Source data are provided as a Source Data file

A central concept in our approach is that HSPCs and their progeny will secrete stable, supra-endogenous IDUA levels that can cross-correct the lysosomal defect in affected cells. Examination of modified HSPCs in culture showed that 3 days post-modification, three distinct cell populations could be discerned based on YFP expression: high/medium/low (Fig. 2a). YFP-high cells exhibited persistent fluorescence in culture for at least 30 days, demonstrating stable integration of the cassettes. YFP-negative cells had no detectable YFP expression at the time of selection, though approximately 1% of cells eventually became positive. Most cells with intermediate fluorescence converted to YFP-high (80%) (Fig. 2b). In these cultures, where YFP-positive and negative cells were mixed and grown under expansion conditions, the fraction of YFP-positive cells remained stable for 30 days, suggesting that neither the modification, nor the overexpression of the enzyme, nor reporter expression in vitro impacted the cells’ proliferative potential.

When compared to mock-treated cells expressing endogenous IDUA levels, YFP-high cells secreted 250 and 25-fold more enzyme for the SFFV and PGK-driven cassettes respectively, while cell lysates expressed 600 and 50-fold more enzymatic activity (Fig. 2c). Not surprisingly, the SSFV promoter was able to drive substantially higher IDUA expression compared to PGK. When YFP-high IDUA-HSPCs were co-cultured with patient-derived MPSI fibroblasts, they led to a decrease in the average area of lysosomal-associated membrane protein 1 (LAMP-1) positive specks, consistent with reduced lysosomal compartment size and cross-correction of the cellular phenotype (Fig. 2d). These data confirm that IDUA-HSPCs secrete supra-physiological IDUA levels and that the secreted IDUA has the post-translational modifications required for uptake into MPSI cells and cellular cross-correction.

For IDUA-HSPCs to successfully correct biochemical abnormalities in the organs affected in MPSI, they must differentiate into monocytes that will migrate to and differentiate into tissue-resident macrophages such as microglia (brain), Kupffer cells (liver), osteoclasts (bone), and splenic macrophages to deliver the enzyme and cross-correct enzyme-deficient cells. To confirm that IDUA-HSPC could generate macrophages and that these cells can continue to produce IDUA, we differentiated these cells in culture and assayed for IDUA activity (Supplementary Note 2). After 3 weeks in a cytokine cocktail containing M-CSF and GM-CSF^23^, cells had macrophage morphology, expressed the macrophage markers CD11b (~89%) and CD14 (~65%), and showed robust phagocytic activity consistent with a macrophage phenotype (Fig. 2e and Supplementary Fig. 3a, b). These IDUA-HPSC-derived macrophages secreted 192-fold and 37-fold more IDUA for the SFFV and PGK-driven cassettes respectively than mock-cell-derived macrophages. Likewise, lysates exhibited 255-fold and 45-fold more IDUA activity (Fig. 2f). These data established that IDUA-HPSC can reconstitute monocyte/macrophages in vitro and that IDUA-HPSC-derived macrophages also exhibit enhanced IDUA expression.

### Preserved repopulation and differentiation in IDUA-HSPCs

**Fig. 3:**
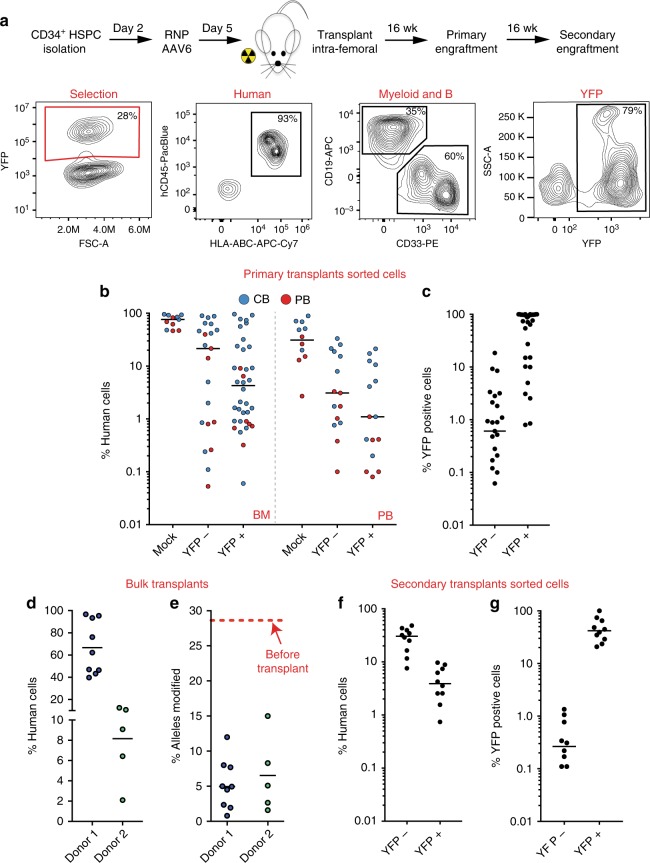
IDUA-HSPCs maintain long-term repopulation capacity. **a** Schematic and representative FACS plots showing phenotyping by flow of human, myeloid, B-cell, and targeted cells after engraftment. **b** Percent human cell chimerism in bone marrow (BM) and peripheral blood (PM) of mice 16-weeks post-transplant with CB (blue dots) and PB (red dots)-derived HSPCs targeted with PGK-IDUA-YFP cassette. Each point represents an individual mouse; mock (*n* = 11), YFP- (*n* = 21), and YFP + (*n* = 36). **c** Percent human, YFP + cells in BM of mice in BM 16-weeks post-transplant. **d** Percent human cell chimerism in BM in mice transplanted with bulk cells without selection with two different human cell donors; donor 1 *n* = 9, donor 2 *n* = 5 **e** Percent modified alleles in engrafted cells by ddPCR. 28% was the starting allele modification frequency for both human donors. **f** Percent human cell chimerism in BM of mice in secondary transplants 32 weeks after genome editing; YFP- (*n* = 10), and YFP+ (*n* = 10). **e** Percent human, YFP+ cells in BM of mice in secondary transplants

To determine if HSPCs that have undergone genome editing can engraft in vivo, we performed serial engraftment studies into NOD-scid-gamma (NSG) mice. We first tested cells modified with the SFFV and PGK constructs expressing YFP, which allowed us to identify the modified cells in vivo. Equal numbers of CB and PB-derived mock, YFP-negative (YFP−), and YFP-positive (YFP+) cells were transplanted intra-femorally into sub-lethally irradiated 6–8-week-old mice. Primary human engraftment was measured 16 weeks-post-transplantation by establishing the percent of bone marrow (BM) cells expressing both human CD45 and human leukocyte antigens (HLA-ABC) out of total mouse and human CD45+ cells (Fig. 3a and Supplementary Fig. 3c). For the PGK-driven constructs, the median frequencies of hCD45^+^/HLA^+^ cells in BM were as follows: Mock 76.25% (min-max: 46.4–95.4%), YFP−21.5% (0.06–89.5%), YFP+ 4.3% (0.06–96%) (Fig. 3b). This showed a 5-fold drop in repopulation capacity in cells that underwent HR-GE (YFP+) compared to cells that did not but were also exposed to RNP, AAV transduction, and sorting (YFP−). The median frequency of human cells expressing YFP was 0.6% (0–18.5%) and 95.8% (1–100%) for YFP− and YFP+ transplants respectively, confirming that edited cells had engrafted in these mice (Fig. 3c). Human cells were also found in the peripheral blood with median frequencies of 31/3.1/1.1% in mock, YFP−, and YFP+ cells respectively (Fig. 3b).

The apparent engraftment advantage of cells that had not undergone HR-GE was also examined by transplanting bulk populations of HSPCs modified with the cassette without YFP. In two independent experiments, an initial fraction of targeted alleles of 28% (43% modified cells) declined to 5.2% and 6.5% in the engrafted cells (8 and 10% modified cells) despite big differences in human chimerism (Fig. 3d, e). This corresponded to a 5-fold drop in donor 1 and 4-fold drop in donor 2. Interestingly, this fall in targeted alleles showed significant variation in individual mice (2 to 10-fold). This data re-demonstrated the observed loss in engraftment potency after modification^17,18,24–26^.

Serial transplantation is considered a gold standard to assess self-renewal capacity of HSCs. For secondary transplants, we isolated human CD34^+^ cells from the bone marrow of primary mice and transplanted into secondary mice. YFP+ engrafted mice showed 3.9% (0.8–9.7%) median human cell chimerism, while YFP- mice showed 30.4% (7.7–48.2%) (Fig. 3f). YFP expression in the engrafted human cells was 0.27% (0–1.35) for YFP- cells, and 41.9% (20.8–100) for YFP+ cells (Fig. 3g). Similar levels of human cell chimerism were observed for the SFFV-driven constructs in serial transplants (Supplementary Fig. 4). Collectively, the presence of YFP-expressing cells at 16- and 32-weeks post-modification demonstrates that cells with long-term repopulation potential can be edited, albeit at lower frequencies than cells that did not undergo HR-GE.

To establish the modified cells’ ability to differentiate into multiple hematopoietic lineages, we looked in vitro using colony formation unit assays (CFUs) and in vivo after engraftment in NSG mice. In CFUs, CB-derived and PB-derived YFP-expressing cells gave rise to all progenitor cells at the same frequencies as mock-treated and YFP- cells, indicating that IDUA-HSPCs can proliferate and differentiate into multiple lineage progenitors in response to appropriate growth factors (Supplementary Fig. 5a, b). In vivo, B, T and myeloid cells were identified using the human CD19, CD3, and CD33 markers. Compared to mock cells that demonstrated a roughly equal distribution of B and myeloid cells (1:1, CD19:CD33) 16-weeks post-transplantation, YFP+ and YFP− cells showed skewing towards myeloid differentiation (YFP+ = 1:16, and YFP− = 1:5) (Supplementary Fig. 5c). Examination of the human cell chimerism vs. percent myeloid content per mouse, revealed that low human engraftment is more likely to be associated with a predominant myeloid population (Supplementary Fig. 5d). This myeloid bias was not observed in circulating cells in the peripheral blood or in secondary transplants (Supplementary Fig. 5e, f). These data suggest that myeloid skewing is inversely correlated with the degree of human cell engraftment, and that neither the genome editing process, nor IDUA expression, affects the modified cell’s capacity to differentiate into multiple hematopoietic lineages in vitro or in vivo.

### IDUA-HSPCs biochemically correct NSG-IDUA^X/X^ mice

**Fig. 4:**
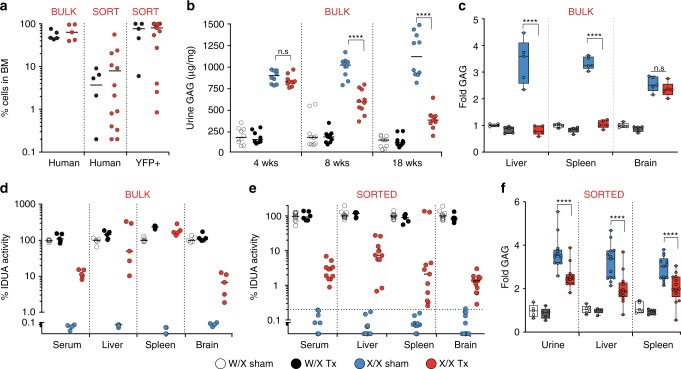
Biochemical correction in NSG-IDUA^X/X^ mice by human IDUA-HSPCs. IDUA activity and GAG accumulation in heterozygous sham-treated (W/X sham- clear), heterozygous transplanted (W/X Tx- black), homozygous sham-treated (X/X sham- blue), and homozygous transplanted (X/X Tx- red) mice. **a** Percent human and YFP + cells in BM in experiments using bulk and sorted cells. **b** Urinary GAGs at 4,8, and 18 weeks in experiments using bulk cells (*n* = 5 mice per cohort, two measurements per mouse). **c**Plasma and tissue IDUA activity in experiments using bulk cells (*n* = 5 per cohort). **d** Fold GAG storage in liver, spleen, and brain (normalized by W/X sham, *n* = 5 per cohort). **e** Plasma and tissue IDUA activity in experiments using sorted cells (*n* = 5 for W/X Tx and sham mice, and *n* = 13 for X/X Tx and sham mice). **f** Fold GAG urinary excretion and tissue storage in experiments using sorted cells (normalized by W/X sham). Median values shown in all scatter plots. **d**, **f** shows box plots with whiskers at the 5–95th percentiles. ****p < 0.0001 in one-way ANOVA test. Post hoc comparisons were made with the Tukey’s multiple comparisons test. Source data are provided as a Source Data file

To determine the potential of human IDUA-HSPCs to correct the metabolic abnormalities in MPSI, we established a new mouse model of the disease capable of engrafting human cells. We used CRISPR-Cas9 to knock-in the W392X mutation, analogous to the W402X mutation commonly found in patients with severe MPSI, into NSG mouse embryos (Supplementary Note 3). Homozygous NSG-IDUA^X/X^ mice replicated the phenotype of patients affected with MPSI^1^ and previously described immunocompetent^27,28^ and immunocompromised^29^ MPSI mice (Supplementary Figs. 6 and 7). We focused the correction experiments on cells expressing IDUA under the PGK promoter, as this promoter has better translational potential because it has decreased enhancer-like activity and less prone to silencing compared to SFFV^30^. In the first series of experiments, we examined PB-derived cells in which the modification did not include a selection marker. In bulk transplants, the median human cell chimerism in the bone marrow was 62.2% (min = 39.2, max = 96.7%) and no statistically significant differences in human engraftment were observed between NSG-IDUA^X/X^ and NSG-IDUA^W/X^ mice (Fig. 4a). GAG urinary excretion was measured at 4, 8, and 18 weeks post-transplantation in NSG-IDUA^X/X^ and IDUA^W/X^ mice. Biochemical correction was detectable after 4 weeks and improved over time (Fig. 4b). This kinetics are consistent with the time lag needed for the genetically engineered HSCs to engraft, expand, and migrate to affected tissues and cross-correct diseased cells. At 18 weeks, NSG-IDUA^X/X^ mice that had been transplanted with IDUA-HSPCs (X/X Tx) excreted 65% less GAGs in the urine compared to sham-treated NSG-IDUA^X/X^ mice (X/X sham) (median Tx = 387.2 µg/mg of creatinine, sham = 1,122 µg/mg) though the levels had not normalized (W/X sham = 155 µg/mg) (Fig. 4b). Transplantation of IDUA-HSPCs also resulted in normalization of tissue GAGs in liver and spleen but not in brain (Fig. 4c). Plasma and brain samples were also analyzed for GAG content and composition by liquid chromatography tandem mass spectrometry (LC-MS/MS)^31^. GAGs species including dermatan sulfate, heparan sulfate and keratan sulfate showed statically significant reductions in the plasma but not in the brain of transplanted NSG-IDUA^X/X^ mice (Supplementary Fig. 8). Notably, plasma keratan sulfate in MPSI is derived from bone damage^32^ not from decreased IDUA activity suggesting improvement in bone dysplasia in the transplanted mice. Transplantation of IDUA-HSPCs also led to increased IDUA activity to 11.3%, 50.1%, 167.5%, and 6.8% of normal in plasma, liver, spleen, and brain respectively (compared to undetectable in X/X sham) (Fig. 4d). In the spleen, supra-endogenous levels of activity were detected consistently and can be attributed to robust human cell engraftment in this organ in the NSG mouse model. Hepatomegaly also significantly improved (Supplementary Fig. 9a–c).

Because we could not discount the contribution of unmodified cells to the observed correction in bulk transplants, we then examined the effect of HSPCs expressing IDUA and YFP under the PGK promoter after FACS-based selection. Of 15 NSG-IDUA^X/X^ and 5 NSG-IDUA^W/X^ mice, 13/15 and 5/5 were deemed to have engrafted (human chimerism in the bone marrow >0.1%). The median percent human chimerism was 4.2% in heterozygous (median percent YFP + 77%) and 9.9% in homozygous mice (median percent YFP + 80%) (Fig. 4a). IDUA-YFP-HSCPs increased IDUA tissue activity to 2.9%, 7.4%, 2.5%, and 1.3% of normal in plasma, liver, spleen, and brain respectively (Fig. 4e). Tissue and urine GAGs were also significantly reduced in spleen and liver (Fig. 4f). Together, this data indicates that IDUA-HSPCs can improve the metabolic abnormalities in MPSI and suggest that the degree of correction correlates with human cell chimerism.

### IDUA-HSPCs phenotypically correct NSG-IDUA^X/X^ mice

**Fig. 5:**
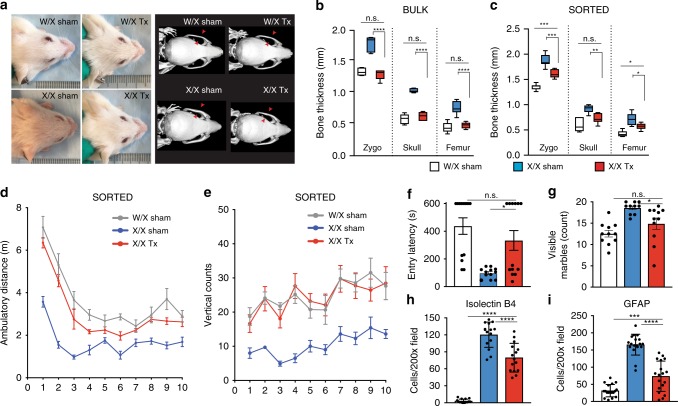
Phenotypic reconstitution in NSG-IDUA^X/X^ mice by human IDUA-HSPCs. **a** Representative photos showing facial features in mice transplanted with bulk cells. **b** Bony features in mice transplanted with bulk cells (W/X sham (clear), X/X sham (blue), and X/X Tx (red), *n* = 5 mice per cohort. Box plots with whiskers show median, min and max. **c** Bony features in mice transplanted with sorted cells (W/X sham (clear or gray, *n* = 11), X/X sham (blue, *n* = 10), and X/X Tx (red, *n* = 11). **d** Ambulatory distance in mice transplanted with sorted cells. W/X sham vs. X/X sham: **; W/X sham vs. X/X Tx: n.s.; X/X sham vs. X/X Tx: *. **e** Vertical rearing in mice transplanted with sorted cells. W/X sham vs. X/X sham: *; W/X sham vs. X/X Tx: n.s.; X/X sham vs. X/X Tx: *. **f** Memory retention in mice transplanted with sorted cells. **g** Quantification of digging behavior in mice transplanted with sorted cells. **h**–**i**. Measurement of neuroinflammation in the cerebral cortex, *n* = 3 mice in five sections. **h** Microglia (Isolectin B4, *n* = 15 brain sections from three independent mice, and **i** astrocytes (GFAP, *n* = 15 brain sections from 3 independent mice. For **d**–**g**, data shown as mean ± SEM. For **h**–**i**, data shown as mean ± SD. All comparisons between groups were performed using one-way ANOVA test and post hoc comparisons were made with the Tukey’s multiple comparisons test. *p < 0.05, **p < 0.01, ***p < 0.001, and ****p < 0.0001. Open field testing and vertical rearings were analyzed using within-subject modeling by calculating the area under the curve for each mouse within the first five minutes and comparing between groups with one-way ANOVA. Source data are provided as a Source Data file

To investigate the effect of IDUA-HSPCs on the skeletal and neurological manifestations of MPSI, sham-treated and transplanted mice also underwent whole body micro-CT and neurobehavioral studies 18 weeks after transplantation. The effect of transplantation on the skeletal system was measured on the skull parietal and zygomatic bone thickness and the cortical thickness and length of femoral bones. In experiments where the mice were transplanted using unselected cells (bulk) and where human cell chimerism was high (Fig. 4a), we observed almost complete normalization of bone parameters by visual inspection and on CT scan measurements (Fig. 5a, b). Mice transplanted with cells that had undergone selection showed partial but statistically significant reduction in the thickness of the zygomatic, parietal bones, and femur (Fig. 5c).

We also examined the open field behavior, passive inhibitory avoidance, and marble-burying behavior of sham-treated and transplanted mice. Transplantation of bulk cells resulted in reduced locomotor activity and long-term memory, regardless of genotype (Supplementary Fig. 10a–c). We suspected that high human-cell chimerism was detrimental for the overall health of the mice. Consistent with this, we observed growth restriction following human cell transplantation in both homozygous and heterozygous mice (Supplementary Fig. 9d). This likely represents a toxicity artifact of this xenogeneic transplant model and could be explained in part by the defective erythropoiesis seen in these xenograft models^33^. In contrast, NSG-IDUA^X/X^ mice transplanted with YFP-selected cells in which human cell chimerism was not as high exhibited locomotor activity indistinguishable from their sham-treated heterozygous littermates, and markedly higher that the sham-treated knock-out mice (Fig. 5d). These mice also had increased vertical counts at all time points and demonstrated the same exploratory behavior as sham heterozygous mice (Fig. 5e). Transplantation of IDUA-HSPCS in NSG-IDUA^X/X^ also enhanced performance in the passive inhibitory avoidance test 24 h later (Fig. 5f). Digging and marble-burying behavior also improved but did not normalize (Fig. 5g).

Because neuroinflammation has been reported in immunocompetent MPSI mice^34^, we looked for effects on brain microgliosis and astrocytosis following transplantation of IDUA-HSPCs. Heterozygous sham-treated (W/X sham), homozygous sham-treated (X/X sham) and homozygous IDUA-HSPC-transplanted (X/X Tx) mouse brains were analyzed 16-week post-transplantation by immunohistochemistry. Microglial activation, assessed by the number of isolectin B4-positive cells^35^ was significantly reduced in X/X Tx compared to X/X sham mice (Fig. 5h and Supplementary Fig. 10d). Astrocyte activation, as measured by the number of Glial fibrillary acidic protein (GFAP) positive astrocytes, was also significantly reduced (Fig. 5i and Supplementary Fig. 10e).

### Safety of our genome editing strategy

**Fig. 6:**
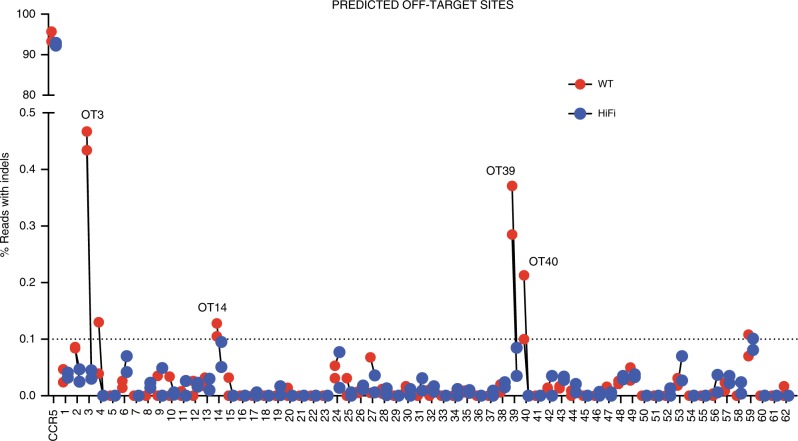
OFF-target analysis of the *CCR5* sgRNA. Percent reads with Indels at 62 off-target sites (OT) predicted using COSMID. For each site, red dots indicate samples treated with WT Cas9 and blue dots indicate samples treated with HiFi Cas9. The limit of detection for NGS is 0.1% and is indicated on the graph by a dashed line

To assess genotoxicity and characterize the off-target repertoire of our *CCR5* guide, we used the bioinformatics-based tool COSMID (CRISPR Off-target Sites with Mismatches, Insertions, and Deletions)^36^. Off-target activity at a total of 67 predicted loci was measured by deep sequencing in two biological replicates of CB-derived HSPCs. In each replicate we compared the percent Indels measured in mock and cells electroporated with RNP with either wild-type (WT) Cas9 or a higher fidelity (HiFi) Cas9^37^. Five of the 67 sites were located within repetitive elements and Indel rates could not be assigned to specific loci in this group (Supplementary Data file). For the remaining 62 genomic locations, sites were deemed true off-targets if: (1) the percent of indels at the site was >0.1% (limit of detection), (2) off-target activity was present in both biological samples, and (3) indels were higher in the RNP compared with the mock samples. Given these criteria only four sites were deemed to be true off-targets (Fig. 6a and Table 1). For all of these sites the frequency of Indels was <0.5% and the use of the HiFi Cas9 abolished off-target activity entirely while maintaining on-target efficiency. Only one exonic site was found in the *SUOX* gene (sulfite oxidase). The highest off-target activity measured at this site was 0.128%, which was reduced below the limit of detection with HiFi Cas9. These data suggest that our *CCR5* sgRNA combined with either WT Cas9 or especially HiFi Cas9 has negligible off-target activity on a large screen of bioinformatically predicted sites.

**Table 1:** Summary of off-target sites (OT’s) above level of detection (>0.1%)

Target	Sequence	Closest gene	Feature	% Reads with Indels				
				Mock	WT1	WT2	HF1	HF2
CCR5	GCAGCATAGTGAGCCCAGAA**GGG**	CCR5	Exon	0.128	93.41	95.832	92.377	93.078
CCR5_OT3	*A*CAGAATAG*A*GAGCCCAGAA**AGG**	GRID1	Intergenic	0	**0.467**	**0.434**	0.03	0.045
CCR5_OT14	*A*CAGCATAG*A*G*G*GCCCAGAA**GGG**	SUOX	Exon	0	**0.105**	**0.128**	0.095	0.051
CCR5_OT39	*A*CAGCATAGTGA*A*CCCAGGA**GGG**	TBPL2	Intergenic	0.017	**0.388**	**0.302**	**0.102**	0.052
CCR5_OT40	GC*T*GCATAGTGA*A*CCCAG*T*A**TGG**	ZNF609	Intergenic	0.032	**0.122**	**0.245**	0.031	0.014

PAM sequences are shown as bold and mismatched bases are shown as italics. For all of these sites the percent of Indels was   <0.5% using wild type (WT) Cas9. For 4 of these sites, the use of the HiFi Cas9 abolished off-target activity

Several studies have shown that in primary cells, Cas9-mediated DSBs result in p53-mediated cell cycle arrest thereby decreasing the efficiency of HR-GE^38,39^. Consequently, concerns have been raised about the potential for enrichment of p53 negative clones when selecting cells that have successfully undergone HR-GE. To examine p53 function in our cells, we targeted four biological samples (two CB and two PB-derived) to express the PGK-IDUA-YFP cassette and separated mock, YFP+ (cells that underwent HR-GE), and YFP− (cells that did not undergo HR-GE). Because p53- clones could be a rare population with a growth advantage, to increase the probability of detection the cells were allowed to expand 100-150-fold (~2 weeks). We first sequenced all *TP53* exons (NM_000546.5) in the three conditions in all four samples using a clinically validated, next-generation sequencing assay. No new TP53 sequence variants were found in the HR-GE+ cells despite in vitro expansion. Consistent with this, after treatment with the DNA double-strand break inducer doxorubicin, mock, HR-GE+, and HR-GE- cells had undisguisable responses when assayed for p53 activation, as measured by p53 protein stabilization by FACs, and transcriptional activation of seven p53 targets genes as measured by qPCR (Supplementary Fig. 11).

Collectively, we performed 200 autopsies (101 mice used in primary engraftment, 50 in secondary engraftment, and 49 in NSG-IDUA^X/X^ correction studies) in which no gross tumors were found. Three tumor-like masses were evaluated by histology and confirmed to be abscesses. These 200 mice were transplanted with a combined dose of 90 million human cells that underwent our genome editing protocol. Considering that the median age for HSCT in MSPI patients is around one year^40^, and that an average one year-old is 10 Kg, the total number of modified cells used in this study is roughly equivalent to two clinical doses of 4.5 × 10^6^ CD34 HSPCs/kg. We conclude that the apparent lack of tumorigenicity and the low off-target activity of the *CCR5* sgRNA provide evidence for the safety our modification strategy.

## Discussion

We describe an efficient application of RNP and AAV6-mediated template delivery to overexpress IDUA from a safe harbor locus in human CD34+ HSPCs. The suitability for *CCR5* to be a safe harbor for the insertion and expression of therapeutic genes has been described^30,41^. For LSDs like MPSI, the use of the safe harbor would have several advantages compared to genetic correction of the affected locus: (1) it enhances potency, as it allows for supra-endogenous expression, (2) it circumvents design for specific mutations in a gene, (3) the coding sequences can be engineered with enhanced therapeutic properties, e.g., crossing the blood brain barrier^42^, (4) it is versatile and easily adaptable to other LSDs, and 5) it avoids the potential risk of uncontrolled integrations (safety).

We studied the self-renewal and multi-lineage differentiation capacity of the modified cells. Our data demonstrates that this approach can modify cells with long-term repopulation potential and preserves multi-lineage differentiation capacity in vivo and in vitro. However, in experiments comparing engraftment potential of the YFP− and YFP+ cells, as well as in bulk transplantation experiments, cells that underwent HR-GE had approximately a 5-fold lower long-term engraftment capacity. Based on observations that HR efficiencies are higher in cycling cells^43,44^, one explanation is that HR happens more readily in the cycling progenitor population that in the more quiescent stem cells. The lower engraftment could also represent a negative effect of expression of a foreign fluorescent protein in HSCs^45^ as previously substituting a truncated form of the low-affinity nerve growth factor receptor resulted in higher engraftment frequencies than using a fluorescent protein to mark HR-GE cells^18^. This is an important caveat for future therapeutic applications, particularly in diseases where high chimerism is required. As observed in allo-HSCT, this engraftment challenge might be partly circumvented by using larger doses of genome-edited cells which can be facilitated by in vitro expansion in optimized culturing conditions that maintain self-renewal capacity^46,47^. Nevertheless, increasing the efficiency of HR-GE in long-term repopulating HSCs will greatly facilitate the clinical application of genome editing in these cells. Current approaches aimed at increasing the efficiency of HR-GE include NHEJ inhibition^48–50^, HR activation^51,52^, limiting p53 pathway activation^53^, cell cycle manipulation^44,54^, and linking the DNA repair template to the genome editing machinery^50,55^.

Ex vivo manipulation of the HSPCs allows for a thorough examination of the genotoxicity and the magnitude of biochemical potency of the cells before delivering the engineered cell product to patients. Through a bioinformatics-guided strategy we identified four potential off-target sites with minimal off-target activity. Fortunately, all but one, were abrogated by using a higher fidelity nuclease^37^. The conclusion that our genome editing strategy is safe is also supported by the lack of tumorigenicity in 200 mice transplanted with 90 million edited cells examined over 16-20 weeks. Furthermore, we showed that HSPCs that have undergone HR-GE and selection had normal p53 function.

Our approach attempts to commandeer the patient’s own hematopoietic system to express and deliver lysosomal enzymes and it is based on clinical experience demonstrating superior outcomes in allo-HSCT compared to ERT particularly in the neurological and musculoskeletal symptoms^56,57^. The autologous source improves on safety, by eliminating the morbidity of graft rejection, graft-versus-host disease, and immunosuppression, and can lead to earlier intervention by obviating the need for donor matching. Compared to non-targeted gene addition to HPSCs using lentiviruses^7,58^, genome editing decreases the potential risks of random viral genome integration and ensures more predictable and consistent transgene expression because the insertion sites are limited to two chromosome loci. Unlike liver-directed approaches using zinc finger nucleases^59^, this approach leverages the unique ability of the hematopoietic system to generate tissue macrophages that can migrate into harder-to-treat organs like the CNS^56,57,60,61^ and, based on clinical experience with allo-HSCT, will provide better correction in the bone and joints. However, like allo-HSCT, autologous transplantation of genetically modified HSPCs might not be sufficient to abolish all musculoskeletal manifestations in humans^62^. While osteoclasts are derived from CD34+ cells and might provide enzyme to correct the bone phenotype, chondrocytes are not derived from these cells.

We examined the potential of the edited HSPCs to reverse symptomatology in a new model of MPSI capable of human cell engraftment. Engraftment of the IDUA-HSPCs led to partial enzyme activity reconstitution in plasma and CNS, and normalization in the liver and spleen. Engraftment also resulted in reductions in GAG storage in multiple organs, except the brain. Notably, small changes in circulating and tissue IDUA lead to significant phenotypic improvements. This is not surprising as even a small fraction of normal IDUA activity can dramatically improve the physical manifestations of MPSI. Mean IDUA activity in fibroblasts from patients with severe MPSI is 0.18% (range 0–0.6), while 0.79% residual activity (range 0.3–1.8) results in mild disease (minimal neurological involvement and the possibility of a normal life span)^63^. In fact, healthy individuals can be found with enzymatic activity as low as 4%^64^. Our data constitutes the first study to show symptomatic correction of a murine model of an LSD with human genome-edited HSPCs and provides support for the further development of this strategy for the treatment of the visceral, skeletal, and neurological manifestations in MPSI.

## Methods

### AAV donor plasmid construction

The CCR5 donor vectors have been constructed by PCR amplification of ~500 bp left and right homology arms for the CCR5 locus from human genomic DNA. SFFV, PGK, IDUA sequences were amplified from plasmids. Primers were designed using an online assembly tool (NEBuilder, New England Biolabs, Ipswich, MA, USA) and were ordered from Integrated DNA Technologies (IDT, San Jose, CA, USA). Fragments were Gibson-assembled into a the pAAV-MCS plasmid (Agilent Technologies, Santa Clara, CA, USA).

### rAAV production

We followed a protocol that has been previously reported with slight modifications^65^. Briefly, HEK 293 cells are transfected with a dual-plasmid transfection system: a single helper plasmid (which contains the AAV rep and cap genes and specific adenovirus helper genes) and the AAV donor vector plasmid containing the ITRs. After 2 days the cells are lysed by three rounds of freeze/thaw, and cell debris is removed by centrifugation. AAV viral particles are purified by ultracentrifugation in iodixanol gradient. Vectors are formulated by dialysis and filter sterilized. Titers are performed using droplet-digital PCR. Alternatively, viruses were amplified and purified by Vigene Biosciences (Rockville, MD, USA).

### Electroporation and transduction of cells

CCR5 sgRNA was purchased from TriLink BioTechnologies (San Diego, CA, USA) and was previously reported^22^. The sgRNA was chemically modified with three terminal nucleotides at both the 5′ and 3′ ends containing 2′ O-Methyl 3′ phosphorothioate and HPLC-purified. The genomic sgRNA target sequence (with PAM in bold) was: CCR5: 5′-GCAGCATAGTGAGCCCAGAAGGG-3′. Cas9 protein was purchased from Integrated DNA Technologies. RNP was complexed by mixing Cas9 with sgRNA at a molar ratio of 1:2.5 at room temperature. CD34^+^ HSPCs were electroporated 2 days after thawing and expansion by using the Lonza Nucleofector 4D (program DZ-100) in P3 primary cell solution as follows: 10 × 10^6^cells/ml, 300 µg/ml Cas9 protein complexed with 150 µg/ml of sgRNA, in 100 µl. Following electroporation, cells were rescued with media at 37 °C after which rAAV6 was added (MOI 15,000 of 15,000 titrated to maximize modification efficiency and cell recovery). A mock-electroporated control was included in most experiments where cells underwent electroporation without Cas9 RNP.

### Quantification of putative CCR5 gRNA off-target activity

Potential off-target sites in the human genome (hg19) were identified and ranked using the recently developed bioinformatics program COSMID^36^, allowing up to three base mismatches without insertions or deletions and two base mismatches with either an inserted or deleted base (bulge). The top ranked sites were further investigated. Off-target activity at a total of 67 predicted loci was measured by deep sequencing in two biological replicates of CB-derived HSPCs. Bioinformatically predicted off-target loci were amplified by two rounds of PCR to introduce adaptor and index sequences for the Illumina MiSeq platform. All amplicons were normalized, pooled and quantified using the PerfeCTa NGS quantification kit per manufacturer’s instructions (Quantabio, Beverly, MA, USA). Samples were sequenced on an Illumina MiSeq instrument using 2 × 250 bp paired end reads. INDELs were quantified as previously described^66^. Briefly, paired-end reads from MiSeq were filtered by an average Phred quality (Qscore) greater than 20 and merged into a longer single read from each pair with a minimum overlap of 30 nucleotides using Fast Length Adjustment of SHort reads. Alignments to reference sequences were performed using Burrows-Wheeler Aligner for each barcode and the percentages of insertions and deletions containing reads within a ±5-bp window of the predicted cut sites were quantified.

### Measuring insertions at the *CCR5* locus with ddPCR

Genomic DNA was extracted from either bulk or sorted populations using QuickExtract DNA Extraction Solution. For droplet-digital PCR (ddPCR), droplets were generated on a QX200 Droplet Generator (Bio-Rad) per manufacturer’s protocol. A HEX reference assay detecting copy number input of the *CCRL2* gene was used to quantify the chromosome 3 input. The assay designed to detect insertions at *CCR5* consisted of: F:5′-GGG AGG ATT GGG AAG ACA -3′, R:5′- AGG TGT TCA GGA GAA GGA CA-3′, and labeled probe: 5′− FAM/AGC AGG CAT /ZEN/GCT GGG GAT GCG GTG G/3IABkFQ-3′. The reference assay designed to detect the *CCRL2* genomic sequence: F:5′-CCT CCT GGC TGA GAA AAA G-3′, R:5′-GCT GTA TGA ATC CAG GTC C-3′, and labeled probe: 5′− HEX/TGT TTC CTC /ZEN/CAG GAT AAG GCA GCT GT/3IABkFQ-3′. The accuracy of this assay was established with genomic DNA from a mono-allelic colony (50% allele fraction) as template. Final concentration of primer and probes was 900 nM and 250 nM respectively. Twenty microliters of the PCR reaction was used for droplet generation, and 40 µL of the droplets was used in the following PCR conditions: 95°–10 min, 45 cycles of 94°–30 s, 57 °C–30 s, and 72°–2 min, finalize with 98°–10 min and 4 °C until droplet analysis. Droplets were analyzed on a QX200 Droplet Reader (Bio-Rad) detecting FAM and HEX positive droplets. Control samples with non-template control, genomic DNA, and mock-treated samples, and 50% modification control were included. Data was analyzed using QuantaSoft (Bio-Rad).

### HSPC selection and culturing

Human CD34+ HSPCs mobilized peripheral blood purchased from AllCells (Alameda, CA, USA) and thawed per manufacturer’s instructions. CD34+ HSPCs were purified from umbilical cord blood collected donated under informed consent via the Binns Program for Cord Blood Research at Stanford University and used without freezing. In brief, mononuclear cells were isolated by density gradient centrifugation using Ficoll Paque Plus. Following two platelet washes, HSPCs were labeled and positively selected using the CD34+ Microbead Kit Ultrapure (Miltenyi Biotec, San Diego, CA, USA) per manufacturer’s protocol. Enriched cells were stained with APC anti-human CD34 (Clone 561; Biolegend, San Jose, CA, USA) and sample purity was assessed on an Accuri C6 flow cytometer (BD Biosciences, San Jose, CA, USA). Cells were cultured at 37 °C, 5% CO_2_, and 5% O_2_ for 48 hours prior to gene editing. Culture media consisted of StemSpan SFEM II (Stemcell Technologies, Vancouver, Canada) supplemented with SCF (100 ng/ml), TPO (100 ng/ml), Flt3-Ligand (100 ng/ml), IL-6 (100 ng/ml), UM171 (35 nM), and StemRegenin1 (0.75 µM).

### Colony forming unit assay and clonal genotyping

Cells were single-cell sorted into 96-well plates (Corning) pre-filled with 100 µl of methylcellulose (Methocult, StemCell Technologies).

Single YFP+, YFP−, and mock-treated cells were sorted into methylcellulose media containing SCF, IL3, erythropoietin, and GM-CSF, conditions that support the growth of blood progenitor cells: erythroid progenitors (burst forming unit-erythroid or BFU-E, and colony-forming unit-erythroid or CFU-E), granulocyte-macrophage progenitors (CFU-GM), and multi-potential granulocyte, erythroid, macrophage, megakaryocyte progenitor cells (CFU-GEMM).

After 14 days, colonies were counted and scored as BFU-E, CFU-M, CFU-GM, and CFU-GEMM per the manual for ‘Human Colony-forming Unit (CFU) Assays Using MethoCult’ from StemCell Technologies. For DNA extraction from 96-well plates, PBS was added to wells with colonies, and the contents were mixed and transferred to a U-bottomed 96-well plate. Cells were pelleted by centrifugation at 300 × *g* for 5 min followed by a wash with PBS. Finally, cells were resuspended in 25 µl QuickExtract DNA Extraction Solution (Epicentre, Madison, WI, USA) and transferred to PCR plates, which were incubated at 65 °C for 10 min followed by 100 °C for 2 min. For *CCR5*, a 3-primer PCR was set up with a forward primer outside the left homology arm (5′-CACCATGCTTGACCCAGTTT-3′), a forward primer binding the poly-adenylation signal in all inserts (5′-CGCATTGTCTGAGTAGGTGT-3′), and a reverse primer binding inside the right homology arm (5′-AGGTGTTCAGGAGAAGGACA-3′). Accupower premix was used for PCR reaction and cycled at the parameters: 95° - 5 min, and 35 cycles of 95° - 20 s, 72 °C – 60 s. DNA fragments were detected by agarose gel electrophoresis.

### Macrophage differentiation and flow cytometry

CD34+ HSPCs were seeded at a density of 2 × 105 cells/mL in untreated 6-well polystyrene plates in differentiation medium (SFEM II supplemented with SCF (200 ng/ml), Il-3 (10 ng/mL), IL-6 (10 ng/mL), FLT3-L (50 ng/mL), M-CSF (10 ng/ml), GM-CSF (10 ng/ml), penicillin/streptomycin (10 U/mL), and cultured at 37 °C 5% CO_2_, and 5% O_2_. After 48 h, non-adherent cells were removed from plates and reseeded in new non-treated 6-well polystyrene plates at 2 × 105 cells/mL in differentiation medium. Adherent cells were maintained in the same plates in maintenance medium (RPMI supplemented with FBS (10% v/v), M-CSF (10 ng/ml), GM-CSF (10 ng/ml), and penicillin/streptomycin (10 U/mL). After three weeks, adherent cells, comprising terminally differentiated macrophages, were harvested by incubation with 10 mM EDTA and gentle scraping. For phenotypic analysis we harvested 1 × 10^5^ cells per condition resuspended in 100 μl staining buffer (PBS containing 2% FBS and 0.4% EDTA). Non-specific antibody binding was blocked (5% v/v TruStain FcX, BioLegend, #422302) and cells were stained with 2 μl of each fluorophore-conjugated monoclonal antibody (30 minutes, 4 °C, dark). Antibodies used were hCD34-APC (BioLegend #343510), hCD14-BV510 (BioLegend #301842) and hCD11b-PE (BioLegend #101208). Propidium Iodide (1 μg/mL)) was used to detect dead cells and cells were analyzed on a BD FACSAria flow cytometer.

### Phagocytosis assay

pHrodo Red *E. coli* BioParticles conjugate for Phagocytosis were purchased from ThermoFisher, USA and reconstituted to 1 mg/mL in 10% FBS-containing media. Reconstituted Bioparticles were added to IDUA-HSPC-derived macrophages and incubated at 37 °C for one hour. The cells were then washed and bathed in imaging media (DMEM Fluorobright, 15 mM HEPES, 5% FBS). Imaging followed using the appropriate absorption and fluorescence emission maxima (560 nm and 585 nm, respectively).

### Mice

NOD.Cg-Prkdc^scid^IL2rg^tmlWjl^/Sz (NSG) mice were developed at The Jackson Laboratory^67^.

Mice were housed in a 12-h dark/light cycle, temperature- and humidity-controlled environment with pressurized individually ventilated caging, sterile bedding, and unlimited access to sterile food and water in the animal barrier facility at Stanford University. All experiments were performed in accordance with National Institutes of Health institutional guidelines and were approved by the University Administrative Panel on Laboratory Animal Care (IACUC 25065).

### Transplantation of CD34^+^ HSPCs into NSG mice

Targeted cells (sorted or bulk) were transplanted four to five days after electroporation/transduction. YFP-negative (YFP-), and YFP-positive (YFP+) cells were isolated using FACS and ~400,000 cells were transplanted intra-femorally into sub-lethally irradiated (2.1 Gy) 6 to 8-week-old mice. Approximately 1 × 10^6^ cells HPSCs modified with cassettes without YFP and were transplanted in bulk. Mice were randomly assigned to each experimental group and analyzed in a blinded fashion.

### Assessment of human engraftment

Sixteen to18 weeks after transplantation, samples of peripheral blood, bone marrow, and spleen were harvested from recipient mice. Samples were treated with ammonium chloride to eliminate mature erythrocytes. Non-specific antibody binding was blocked (10% vol/vol, TruStain FcX, BioLegend), cells were stained (30 min, 4 °C, dark), and analyzed by setting nucleated cell scatter gates using a BD FACSAria II flow cytometer or BD FACSCanto II analyzer (BD Biosciences). Cells were analyzed based on monoclonal anti-human HLA-ABC APC-Cy7 (W6/32, BioLegend), anti-mouse CD45.1 PE-Cy7 (A20, eBioScience, San Diego, CA, USA), CD19 APC (HIB19, BD511 Biosciences), CD33 PE (WM53, BD Biosciences), anti-mouse mTer119 PE-Cy5 (TER-119, BD Biosciences), and CD3 PerCP/Cy5.5 (HiT3A, BioLegend) antibodies, and Propidium Iodide to detect dead cells. Human engraftment was defined as HLA-ABC^+^/HCD45^+^ cells. See Supplementary Methods for a complete Antibody list.

### IDUA activity assay

IDUA enzyme activity was measured fluoremetrically using 4-methylumbelliferyl α-L-iduronide (4MU-iduronide) (LC Scientific Inc., Canada) per established assay conditions^68^. Briefly, for IDUA the 4-methylumbelliferyl-iduronide substrate is diluted with sodium formate buffer, 0.4 M, pH 3.5, to 6.6 mM concentration. Twenty-five microliters of aliquots of substrate are mixed with 25 μL of cell or tissue homogenates and adjusted to a final substrate concentration of 2.5 mM. The mixture is incubated at 37 °C for 60 min, and 200 μL glycine carbonate buffer (pH 10.4) is added to quench the reaction. 4-MU (Sigma) is used to make the standard curve. The resulting fluorescence is measured using a SpectraMax M3 plate reader with excitation at 355 nm and emission at 460 nm (Molecular devices).

### Analysis of GAGs using the DMB method

Urine and tissue GAGs were measured with the modified dimethylmethylene blue assay (DMB)^69^. Tissue samples (10–30 mg) were incubated for 3 h at 65 °C in papain digest solution (calcium- and magnesium-free PBS containing 1% papain suspension (Sigma), 5 mM cysteine, and 10 mM EDTA, pH 7.4) to a final concentration of 0.05 mg tissue/mL buffer. Fifty microliters of extract was incubated with 200 µL DBM reagent (9:1 31 μM DMB stock (in formiate buffer 55 nM): 2 M Tris base). The samples were read on a microplate reader at 520 nm.

### Analysis of GAGs by LC/MS-MS

Disaccharides were produced from polymer GAGs by digestion with chondroitinase B, heparitinase, and keratanase II, resulting in DS (di-0S), HS (diHS-NS, diHS-0S), and KS (mono-sulfated KS, di-sulfated KS). Chondrosine was used as an internal standard (IS). Unsaturated disaccharides, [ΔDiHS-NS, ΔDiHS-0S, ΔDi-4S, mono-sulfated KS and di-sulfated KS were obtained from Seikagaku Corporation (Tokyo, Japan) and used to make standard curves. Stock solutions ΔDiHS-NS (100 μg/ml), ΔDiHS-0S (100 μg/ml), ΔDi-4S (250 μg/ml), mono- and di-sulfated KS (1000 μg/ml) and IS (5 mg/ml) were prepared separately in milliQ water. Standard working solutions of ΔDiHS-NS, ΔDiHS-0S, ΔDi-4S (7.8125, 15.625, 31.25, 62.5, 125, 250, 500, and 1000 ng/ml), and mono- and di-sulfated KS (80, 160, 310, 630, 1250, 2500, 5000, and 10,000 ng/ml) each mixed with IS solution (5 μg/ml) were prepared. Mass spectrometer apparatus, run condition, brain homogenate preparation, and disaccharide analysis were done as described in Supplementary Methods.

### Histology

After bleeding, brains were trans-cardially perfused with Phosphate-buffered saline (PBS, pH 7.4) followed by 4% paraformaldehyde (PFA) in PBS. Brains were fixed overnight at 4 °C. Subsequently, brains were transferred to a 30% sucrose solution overnight for cryoprotection, embedded in Tissue-Tek OCT compound, and cut (15–20 μm sections) on a freezing cryostat (Leica, CM3050). All tissue was stored at −80 °C until further use. For immunohistochemistry, slides were washed in PBS to remove excess OCT. Sections were blocked in 10% normal goat plasma (NGS; Gibco) containing 0.25–3% Triton X-100 for 1 h at 25 °C. Primary antibody (anti-GFAP, 1:500) was applied overnight in 10% NGS with 0.1% Triton X-100 at 4 °C followed by the appropriate fluorochrome conjugated secondary antibody (Alexa conjugates; Molecular Probes) for 1 h at 25 °C. For imaging microglia, Isolectin GS-IB_4_ From *Griffonia simplicifolia*, Alexa Fluor 568 Conjugate (Invitrogen-Molecular Probes, USA) was reconstituted as a 1 mg/ml stock in PBS with 0.5 mM CaCl_2_ and 0.01% sodium azide and the slides were incubated with a working solution of 5 µg/ml in calcium-containing PBS for one hour at 25 °C. Slides were then washed in PBS with 0.1% BSA, counterstained with Hoechst, and mounted in Aqua Poly/Mount (Polysciences, Inc.) for fluorescent microscopy. Slides were visualized by conventional epifluorescence microscopy using an all-in-One Fluorescence Microscope BZ-X800 (Keyence, Itasca, USA).

### Immunocytochemistry

MPSI fibroblasts cells (Coriell Cell Repository, GM000798) were fixed in 4% paraformaldehyde in phosphate-buffered saline (PBS), blocked with 3% bovine serum albumin (BSA) in PBS, and stained with rabbit anti-LAMP1 (Abcam, ab24170, 1:200) followed by 1:500 dilutions of Alexa 488-conjugated anti-rabbit antibody (Molecular Probes). Mounting and staining of nuclei was done Vectashield with DAPI (Vector labs). Slides were visualized by conventional epifluorescence microcopy using a cooled CCD camera (Hamamatsu) coupled to an inverted Nikon Eclipse Ti microscope. Images were acquired using NIS elements software and analyzed with ImageJ.

### Computerized tomography

High-resolution Micro-CT scans were acquired at Stanford Center for Innovation in In Vivo Imaging (SCI^3^) using an eXplore CT 120 scanner (TriFoil imaging). Mice were anesthetized with isoflurane (Baxter Corporation, Mississauga, ON, Canada). The scans were obtained with voxel resolution of 100 μm, an energy level of 80 keV, and 360 degrees of whole mice. Microview software (Parallax innovations) was used for isosurface rendering and measurements. Skull thickness was quantified on Midsagittal images. Femur length was determined by measuring the long axis between the two epiphyses. Zygomatic bone thickness was measures on coronal sections, perpendicular to the axis of the zygoma. Bone lengths were determined using the line measurement tool in MicroView. Femurs were measured from the base of the lateral femoral condyle to the tip of the greater trochanter.

### Spontaneous locomotor activity

All behavioral experimenters were blind to the genotype of the mice throughout testing. All tests were conducted in the light cycle. In all experiments, animals were habituated to the testing room 2 h before the tests and were handled by the experimenter for 3 days before all the behavioral tests. For spontaneous locomotor activity, assessment took place using the open field test in a square arena (76 × 76 cm^2^) with opaque white walls, surrounded with privacy blinds to eliminate external room cues. Mice were placed in the center of the open-field arena and allowed to freely move for 10 min while being tracked by Ethovision (Noldus Information Technology, Wageningen, the Netherlands) automated tracking system. Before each trial, the surface of the arena was cleaned with Virkon disinfectant. For analysis, the arena was divided into a central (53.5 × 53.5 cm^2^) and a peripheral zone (11.25-cm wide).

### Passive inhibitory avoidance

The passive inhibitory avoidance test was used to assess fear-based learning and memory. We used a dual-compartment system (GEMINI system, San Diego Instruments), where lighted and dark compartments, equipped with grid floor that can deliver electrical shocks, are separated by an automated gate. On day one, each mouse was habituated to the apparatus by placing it into the lighted compartment. After 30 s, the gate opened allowing access to the dark compartment. When the mice entered the dark compartment, the gate closed and the time to cross after the gate opened is recorded (latency time). On day 2 or training day, the mice receive a 0.5 mA shock for 2 s after a 3 s delay after crossing from the lighted to the dark compartment. On day 3, or testing day, after being placed in the lighted compartment for 5 s, the gate opened allowing access to the dark compartment. The latency to enter the dark compartment was recorded. Maximum time to cross was 10 minutes.

### Marble burying

Repetitive behavior was tested in the marble bury test. Individual mice were introduced into cages containing 20 black glass marbles (1.5 cm diameter, four equidistant rows of five marbles each) on top of bedding 5 cm deep. After 30 min under low-light conditions, mice were removed and the number of marbles that were at least half-covered was determined.

### NSG-IDUA^X/X^ mice

We used CRISPR-Cas9 to knock-in the W401X mutation (UniProtKB - Q8BMG0), analogous to the W402X mutation commonly found in patients with severe MPSI, into NSG mouse embryos. The guide RNA target sequence was searched using crispr.mit.edu and six shortlisted guides close to the target site were first screened by using an in vivo assay in NIH 3T3 cells. Two guides, one each on both sides of the target site, were selected: Guide1 (5′-TTATAGATGGAGAACAACTC-3′) cleaves 4 bases upstream and Guide3 (5′-GTTGGACAGCAATCATACAG-3′) cleaves 44 bases downstream of the target site. The guides were prepared by in vitro transcription (HiScribe™ T7 High Yield RNA Synthesis Kit, E2040S, New England Biolabs) of a dsDNA template generated by annealing two oligos (with a T7 promoter in the sense oligo) followed by a standard PCR reaction. The ssODN donor DNA contained an intended point mutation leading to a STOP codon (TGG to TAG): 5′-ggtgggagctagatattagggtaggaagccagatgctaggtatgagagagccaacagcctcagccctctgcttggcttatagATGGAGAACAA/CTCT**A**GGCAGAGGTCTCAAAGGCTGGGGCTGTGTTGGACAGCAATCATA/CAGTGGGTGTCCTGGCCAGCACCCATCACCCTGAAGGCTCCGCAGCGGCCTGGAGTAC-3′ (lower case is intron, upper case is exon, guide cut sites marked by “/” and the mutation in bold).

Mouse Zygotes were obtained by mating NSG stud males with super-ovulated NSG females. Female NSG mice 3–4 weeks of age (JAX Laboratories, stock number 005557) were super-ovulated by intraperitoneal injection with 2.5IU pregnant mare serum gonadotropin (National Hormone & Peptide Program, NIDDK), followed 48 hours later by injection of 2.5 IU human chorionic gonadotropin (hCG, National Hormone & Peptide Program, NIDDK). The animals were sacrificed 14 h following hCG administration and fertilized eggs were collected. CRISPR Injection mixture was prepared by dilution of the components into injection buffer (5 mM Tris, 0.1 mM EDTA, pH 7.5) to obtain the following concentrations: 10 ng/μl Cas9 mRNA (Thermo Fisher Scientific, Carlsbad, CA), 10 ng/μl IDUA1F and IDUA3F guide RNA and 10 ng/μl ssODN Donor (Integrated DNA Technologies, Coraville, IA). Zygote injections and embryo transfers were performed using standard protocols^70^. A total of 38 zygotes were injected, the surviving 27 zygotes were transferred, which yielded seven live offspring. Among these a male homozygous for the mutation was used to establish the NSG-IDUA^X/X^ colony. Mice were genotyped by-PCR based amplification followed by Sanger sequencing using the following primers: GENO F: 5′-CATGGCCCTGTTGGGTGAGTAATGA-3′, and GENO R: 5′-TGTGGTACTCCAGGCCGCTG-3′.

### Measurement of p53 protein stabilization by FACs

Human HPSCs were incubated in doxorubicin (Sigma) at 0.2 µg/ml for 6 h. After harvesting and washing with PBS, the cells were incubated with LIVE/DEAD fixable blue dead cell stain for 15 min (ThermoFisher, USA). The cells were fixed with 2% paraformaldehyde for 10 min at RT, and permeabilized with 0.1% Triton X-100 in PBS for 10 min at RT. Cells were blocked using 2% goat serum and 0.5% BSA in permeabilization buffer (15 min at RT) and stained with PE-labeled anti-p53 antibody (clone DO-7, Biolegend, USA) or its isotype control for 1 h at RT in the dark. Flow cytometry data was acquired with FACSAria using FACSDiva software (BD Biosciences).

### *TP53* gene sequencing

Sequencing of samples was performed at the Stanford Molecular Genetic Pathology Clinical Laboratory using a clinically validated, targeted next generation sequencing (NGS) assay. Acoustic shearing of isolated genomic DNA (M220 focused ultrasonicator, Covaris, Woburn, MA) is followed by preparation of sequencing libraries (KK8232 KAPA LTP Library Preparation Kit Illumina Platforms, KAPABiosystems, Wilmington, MA), and hybridization-based target enrichment with custom-designed oligonucleotides (Roche NimbleGen, Madison, WI). The panel covers, partially or fully, 164 genes that are clinically relevant in hematolymphoid malignancies, including *TP53*. Pooled libraries are sequenced on Illumina sequencing instruments (MiSeq or NextSeq 500 Systems, Illumina, San Diego, CA). Sequencing results are analyzed with an in-house developed bioinformatics pipeline. Sequence alignment against the human reference genome hg19 is performed with BWA in paired end mode using the BWA-MEM algorithm and standard parameters. Variant calling is performed separately for single nucleotide variants (SNVs), insertions and deletions < 20 bp (Indels), and fusions. VarScan v2.3.6 is used for calling SNVs and Indels, and FRACTERA v1.4.4 is used for calling fusions. Variants are annotated using Annovar and Ensembl reference transcripts. The assay can detect variants with a variant allele fraction as low 5%.

### qPCR for p53 target genes

RNA collection was performed using RNeasy Mini kit (Qiagen, 74104) according to the manufacturer’s instructions. RNA (2–7 µg) was treated with DNAse I (Invitrogen DNA-free, AM1906) according to the manufacturer’s instructions. Reverse transcription was performed with M-MLV reverse transcriptase (Invitrogen, 28025) and random primers (Invitrogen, 48190). 1 µg of total RNA was used for cDNA synthesis. All samples within an experiment were reverse transcribed at the same time, the resulting cDNA diluted 1:5 in nuclease-free water and stored in aliquots at –80 °C until used. Quantitative PCR was performed in triplicate using PowerUP SYBR green master mix (Life Technologies, A25743) and a 7900HT Fast Real-Time PCR machine (Applied Biosystems). Expression analysis was performed using specific primers for each gene (Table X). The mean of housekeeping gene *HPRT* was used as an internal control to normalize the variability in expression levels. All qRT-PCR performed using SYBR Green was conducted at 50 °C for 2 min, 95 °C for 10 min, and then 40 cycles of 95 °C for 15 s and 60 °C for 1 min. The specificity of the reaction was verified by melt curve analysis. A standard curve was used to quantify the samples. See Supplementary Methods for the list of qRT-PCR primers.

### Statistical analysis

All statistical test including paired and unpaired t-tests, and one-way analysis of variance (ANOVA) followed by Tukey’s multiple comparisons test was performed using GraphPad Prism version 7 for Mac OS X, GraphPad Software, La Jolla California USA. Data was reported as means when all conditions passed three normality tests (D’Agostino & Pearson, Shapiro-Wilk, and KS normality test).

### Reporting summary

Further information on research design is available in the Nature Research Reporting Summary linked to this article.

## Supplementary information

Supplementary Information

Reporting Summary

Description of Additional Supplementary Files

Supplementary Data 1

Source Data

## Data Availability

Data supporting the findings of this work are available within the paper and its Supplementary Information files. All unmodified reads for the sequencing-based data in the manuscript are available from the NCBI Bioproject, accession number PRJNA558781. The datasets generated and analyzed during the current study are available from the corresponding author upon request. The source data underlying Figs. 2b–f, 4c and f, and 5b–i, as well as Supplementary Figs. 2b, 5b, 6a–g, 7a–g, 9a–d and 11a–b are provided as a Source Data file.

## Code availability

To quantitate InDels from Paired End sequenced (Illumina) reads we used the custom script: https://github.com/piyuranjan/NucleaseIndelActivityScript/blob/master/indelQuantificationFromFastqPaired-1.0.1.pl
