# Supplementary material for: Human genome-edited hematopoietic stem cells phenotypically correct Mucopolysaccharidosis type I
Source: Nat Commun. 2019 Sep 6;10:4045. doi: 10.1038/s41467-019-11962-8 (PMC6731271; doi:10.1038/s41467-019-11962-8)
Supplement: Supplementary file 1 — Supplementary Information [file 41467_2019_11962_MOESM1_ESM.pdf]

Supplementary Information:

Human genome-edited hematopoietic stem cells phenotypically correct Mucopolysaccharidosis type I

Gomez-Ospina et, al.

Table of contents: **Page**

Supplementary Figures

Supplementary Figure 1.. 3
Supplementary Figure 2.. 4
Supplementary Figure 3.. 5
Supplementary Figure 4.. 6
Supplementary Figure 5.. 7
Supplementary Figure 6.. 8
Supplementary Figure 7.. 9
Supplementary Figure 8.. 10
Supplementary Figure 9.. 11
Supplementary Figure 10.. 12
Supplementary Figure 11.. 13

Supplementary Notes

Supplementary Note 1.. 14
Supplementary Note 2.. 14
Supplementary Note 3.. 15

Supplementary Tables

Supplementary Table 1... 16

Supplementary Methods…... 17

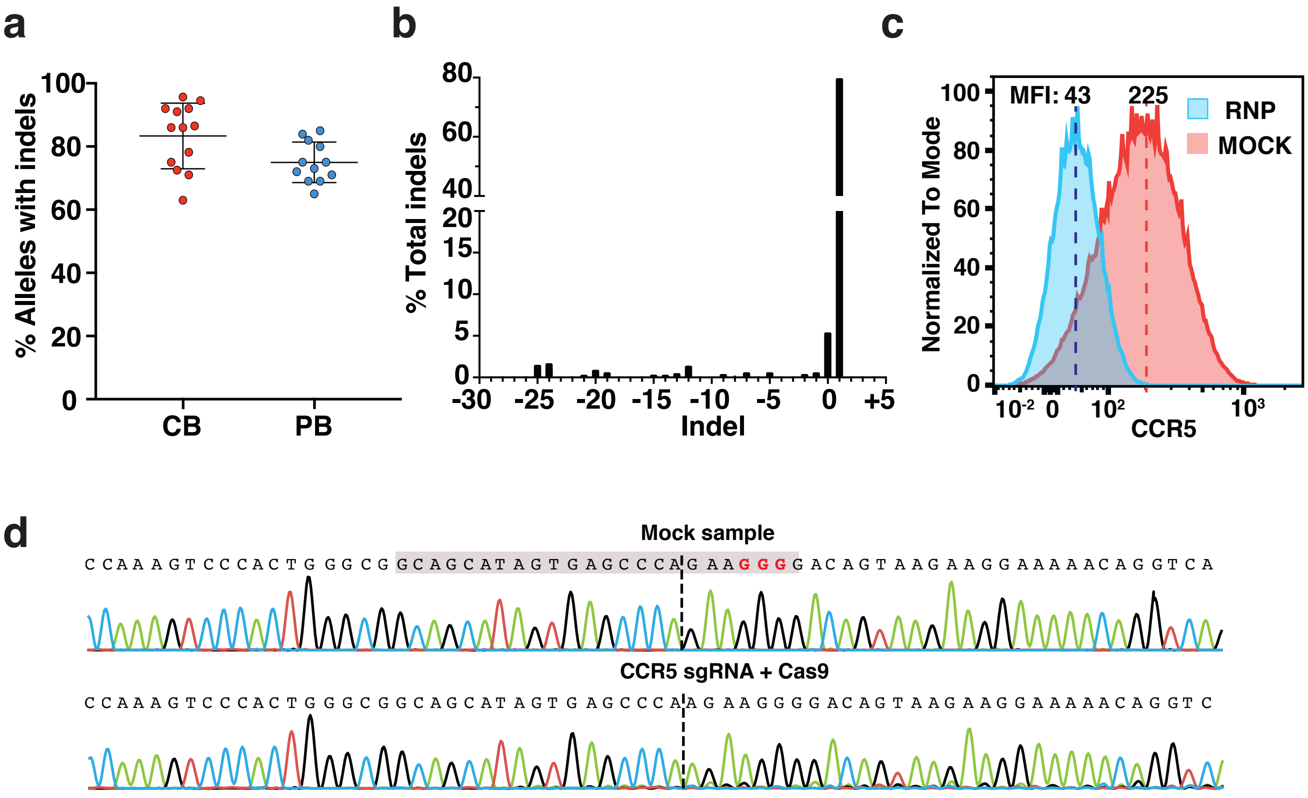

e

Freq (%)	Indel	Sequence
71.27	+1bp	ATTGTATTTCCAAAGTCCCACTGGGCGGCAGCATAGTGAGCCCAAGAAGGGGACAGTAAGAAGGAAAAACAGGTCAGAG
20.90	0	ATTGTATTTCCAAAGTCCCACTGGGCGGCAGCATAGTGAGCCCA-GAAGGGGACAGTAAGAAGGAAAAACAGGTCAGAG
0.78	+2bp	ATTGTATTTCCAAAGTCCCACTGGGCGGCAGCATAGTGAGCCCAAAGAAGGGGACAGTAAGAAGGAAAAACAGGTCAGA
0.68	+1bp	ATTGTATTTCCAAAGTCCCACTGGGCGGCAGCATAGTGAGCCCACGAAGGGGACAGTAAGAAGGAAAAACAGGTCAGAG
0.40	-12bp	ATTGTATTTCCAAAGTCCCACTGGGCGGCAGCATA------------GGGGACAGTAAGAAGGAAAAACAGGTCAGAGA
0.36	-8bp	ATTGTATTTCCAAAGTCCCACTGGGCGGCAGCATAGTGA---------GGGGACAGTAAGAAGGAAAAACAGGTCAGAGA
0.32	-5bp	ATTGTATTTCCAAAGTCCCACTGGGCGGCAGCATAGTGA-----GAAGGGGACAGTAAGAAGGAAAAACAGGTCAGAGA
0.30	+1bp	ATTGTATTTCCAAAGTCCCACTGGGCGGCAGCATAGTGAGCCCCAGAAGGGGACAGTAAGAAGGAAAAACAGGTCAGAG
0.29	-7bp	ATTGTATTTCCAAAGTCCCACTGGGCGGCAGCATAGTG-------AAGGGGACAGTAAGAAGGAAAAACAGGTCAGAGA
0.29	-3bp	ATTGTATTTCCAAAGTCCCACTGGGCGGCAGCATAGTGAGCCCA---GGGGACAGTAAGAAGGAAAAACAGGTCAGAGA
0.29	-2bp	ATTGTATTTCCAAAGTCCCACTGGGCGGCAGCATAGTGAGCCCA--AGGGGACAGTAAGAAGGAAAAACAGGTCAGAGA
0.25	-9bp	ATTGTATTTCCAAAGTCCCACTGGGCGGCAGCATA----------GAAGGGGACAGTAAGAAGGAAAAACAGGTCAGAGA
0.19	+4bp	ATTGTATTTCCAAAGTCCCACTGGGCGGCAGCATAGTGAGCCCAGTAAGAAGGGGACAGTAAGAAGGAAAAACAGGTCA
0.17	-1bp	ATTGTATTTCCAAAGTCCCACTGGGCGGCAGCATAGTGAGCCCAG-AGGGGACAGTAAGAAGGAAAAACAGGTCAGAGA
0.17	+2bp	ATTGTATTTCCAAAGTCCCACTGGGCGGCAGCATAGTGAGCCCACAGAAGGGGACAGTAAGAAGGAAAAACAGGTCAGA
0.17	+1bp	ATTGTATTTCCAAAGTCCCACTGGGCGGCAGCATAGTGAGCCCAGTAAGGGGACAGTAAGAAGGAAAAACAGGTCAGAG
0.15	-10bp	ATTGTATTTCCAAAGTCCCACTGGGCGGCAGCATAGTGAGCCC----------AGTAAGAAGGAAAAACAGGTCAGAGA
0.15	+1bp	ATTGTATTTCCAAAGTCCCACTGGGCGGCAGCATAGTGAGCTCCAGAAGGGGACAGTAAGAAGGAAAAACAGGTCAGAG
0.13	+3bp	ATTGTATTTCCAAAGTCCCACTGGGCGGCAGCATAGTGAGCCCAGGAGAAGGGGACAGTAAGAAGGAAAAACAGGTCAG
0.13	+1bp	ATTGTATTTCCAAAGTCCCACTGGGCGGCAGCATAGTGAGCCGCAGAAGGGGACAGTAAGAAGGAAAAACAGGTCAGAG
0.10	-11bp	ATTGTATTTCCAAAGTCCCACTGGGCGGCAGCATAGTGAG-----------ACAGTAAGAAGGAAAAACAGGTCAGAGA

Supplementary Figure 1 | Characterization of the *CCR5* sgRNA. a, Indel frequency in Cord blood (CB, red) and adult peripheral blood (PB, blue)-derived cells by the RNP complex. Data expressed as mean ± SD. CB n=13 and PB n=13. Each dot represents a different biological sample. **b,** Representative indel distribution from next generation sequencing reads. **c,** Representative histogram of CCR5 protein expression in mock and RNP-treated cells showing an 80% reduction in protein expression after RNP electroporation. **d,** Sample sequence traces around the CCR5 sgRNA sequence (gray box, PAM in red) in mock samples and RNP-treated CB-derived HSPCs showing predominant single A insertion. **e,** Representative summary of indels with frequencies greater than 0.1%.

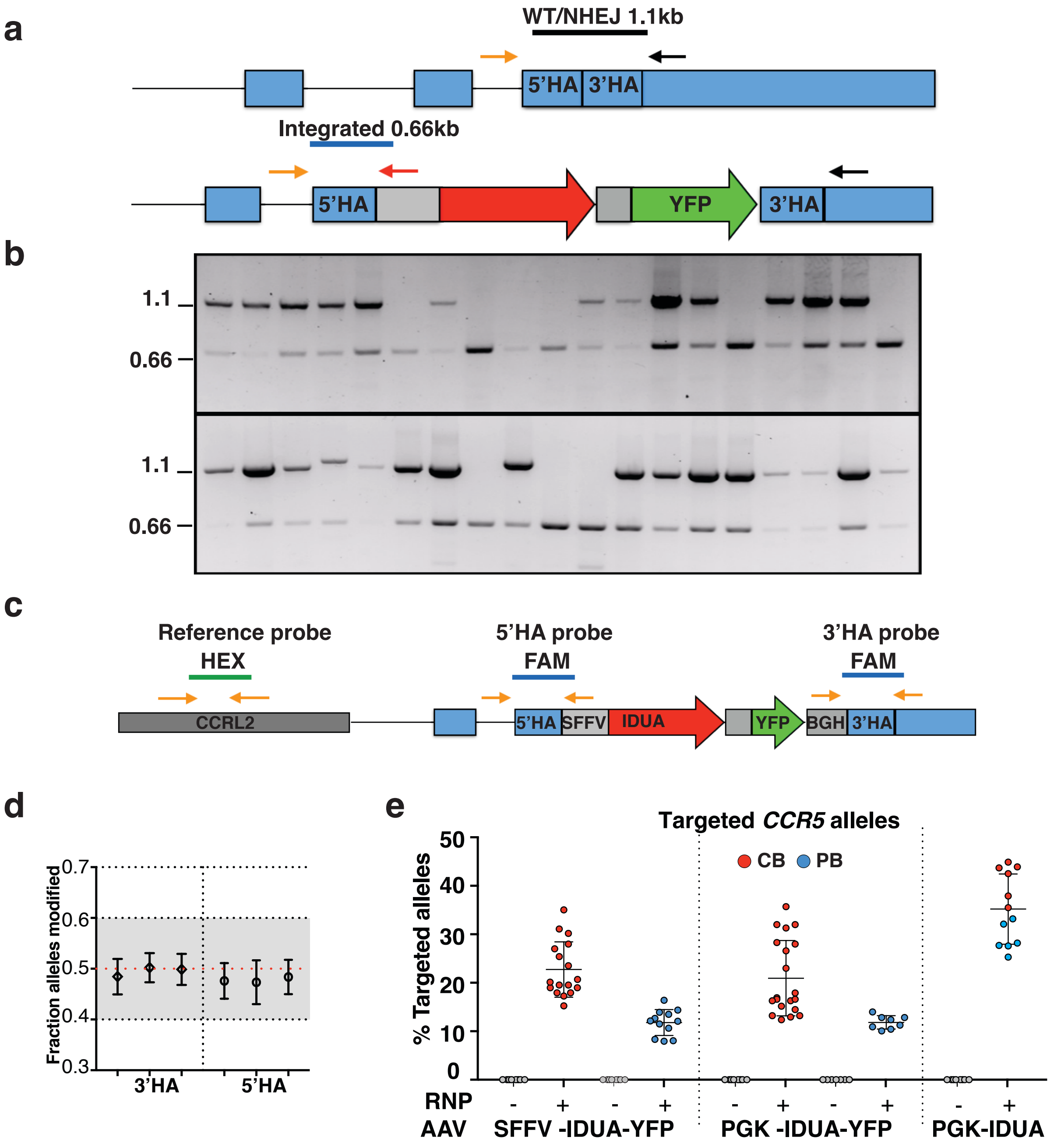

Supplementary Figure 2 | Efficiency of modification at the *CCR5* locus. a, Schematic showing the three primer-based genotyping scheme to distinguish mono and bi-allelic integration into the *CCR5* locus on CFA-derived colonies. This strategy did not distinguish WT versus alleles with indels (NHEJ). **b,** Example agarose gels of 40 colonies genotyped in this manner. A single 1.1Kb band was interpreted as WT/NHEJ in both alleles, while a single 0.6 Kb band was read as bi-allelic integration. **c,** Schematic of probe design for ddPCR analysis. Fraction of modified alleles was obtained by using a second reference probe to the *CCRL2* gene also on chromosome 3p. **d,** Two probes where each straddled a 5' or 3' homology arm were designed. The accuracy of the assays was compared using genomic DNA from colonies derived from mono-allelic cells (0.5 fraction of alleles modified). Error bars indicate 95% CI. The 3' HA probe was selected. **e,** CCR5 allele targeting frequencies in CB (red n=20) and PB (blue n=11)-derived IDUA expressing HSPCs as measured by ddPCR. For the template without selection CB=6, PB=6. Data shown as mean ± SD. Source data are provided as a Source Data file.

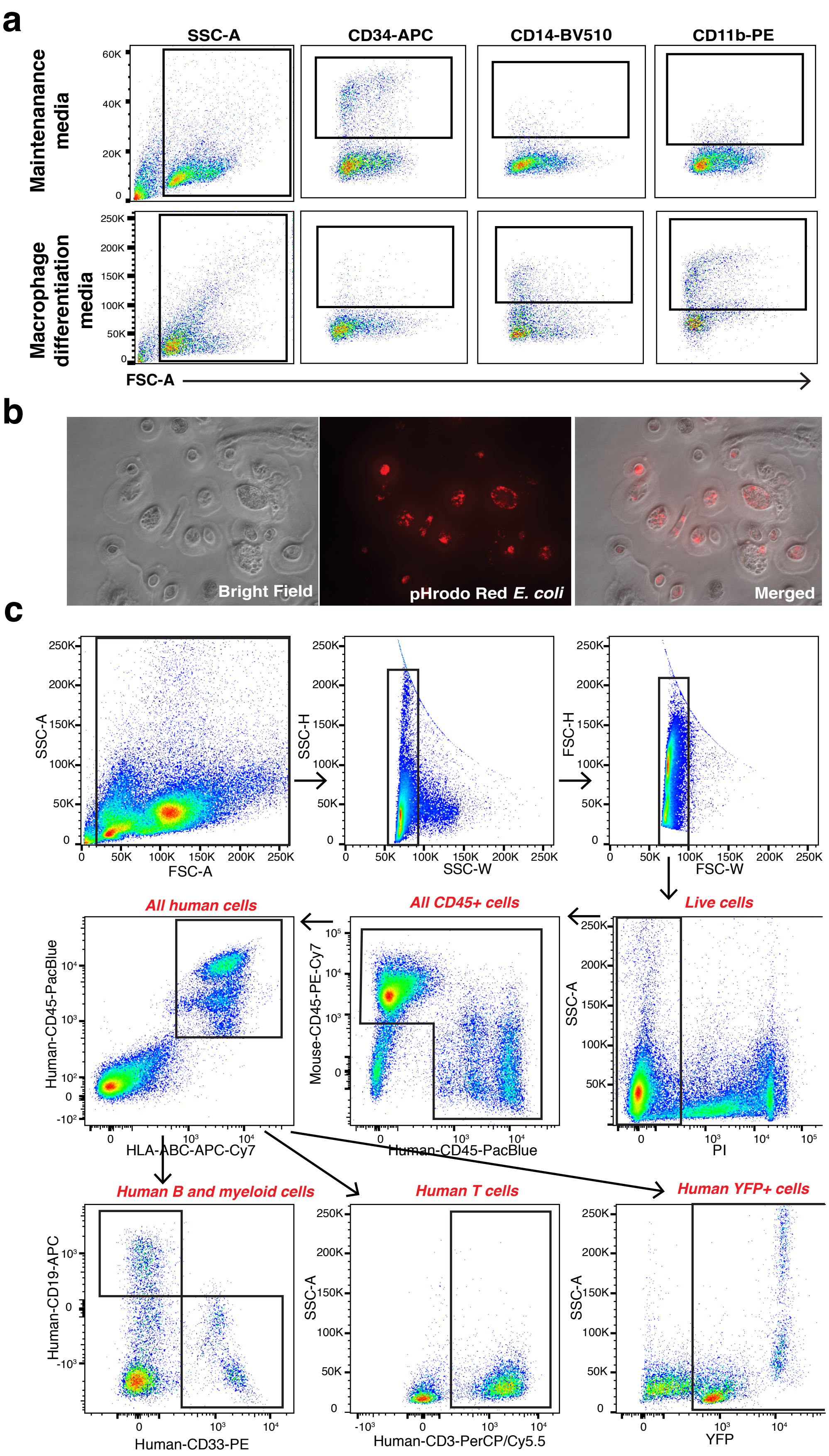

Supplementary Figure 3 | Gating schemes. a, Gating scheme for quantification of human CD34+, CD14+, and CD11b+ cells in human HSPCs maintained in standard CD34+ cytokine media (top) or macrophage differentiation (bottom). Single and live cell discrimination not shown. This strategy was applied to main Figure panel 2e. **b,** Representative images of phagocytosis assay showing HSCP-derived macrophages 3 weeks post-differentiation after incubation with *E. coli* conjugated with a pH sensitive dye (pHrodo). Bright red puncta represent phagosomes. **c,** Gating scheme used to analyze human cell engraftment and cell lineages after transplantation. Representative plots for quantification of mouse and human hematopoietic (mCD45+ and hCD45+), all human (CD45+/HLA-ABC+), human B (CD19+), human myeloid (CD33+), human T (CD3+), and YFP+ cells. This strategy was applied to main figure panels 3b-g and Supplementary Figure 4.

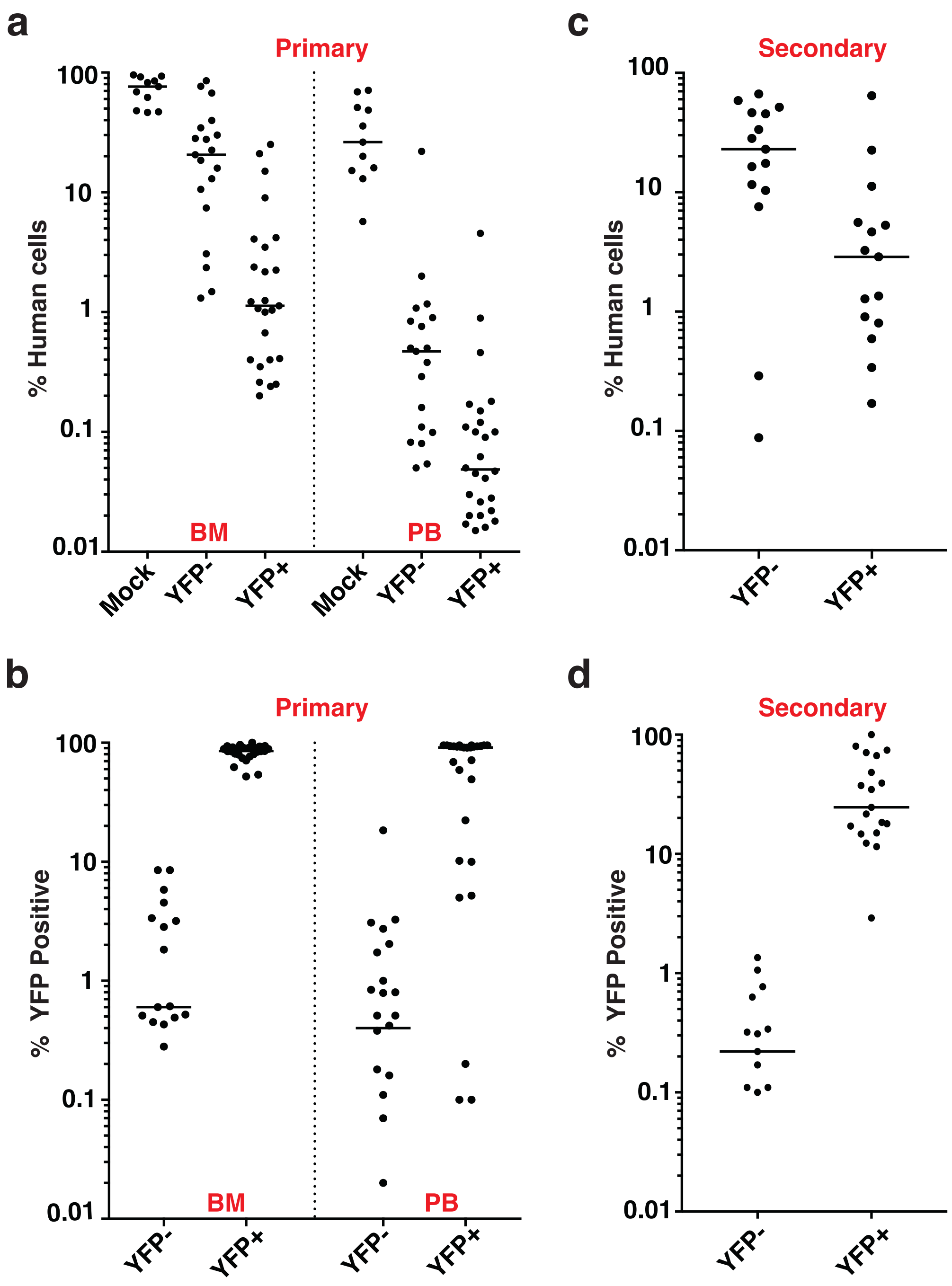

Supplementary Figure 4 | IDUA-HSPCs modified with SFFV containing cassettes are capable of long-term engraftment. a, Percent human cell chimerism in bone marrow (BM) and peripheral blood (PM) of mice 16-weeks post-transplant with CB-derived HPSCs. Each point represents a mouse; Mock (n=11), YFP- (n=19), and YFP+ (n=25). **b,** Percent YFP+ cells in BM and PB of mice in primary transplants. **c,** Percent human cell chimerism in BM of mice in secondary transplants (32 weeks); YFP- (n=15), and YFP+ (n=15). **d,** Percent YFP+ cells in BM and PB of mice in secondary transplants. Medians shown.

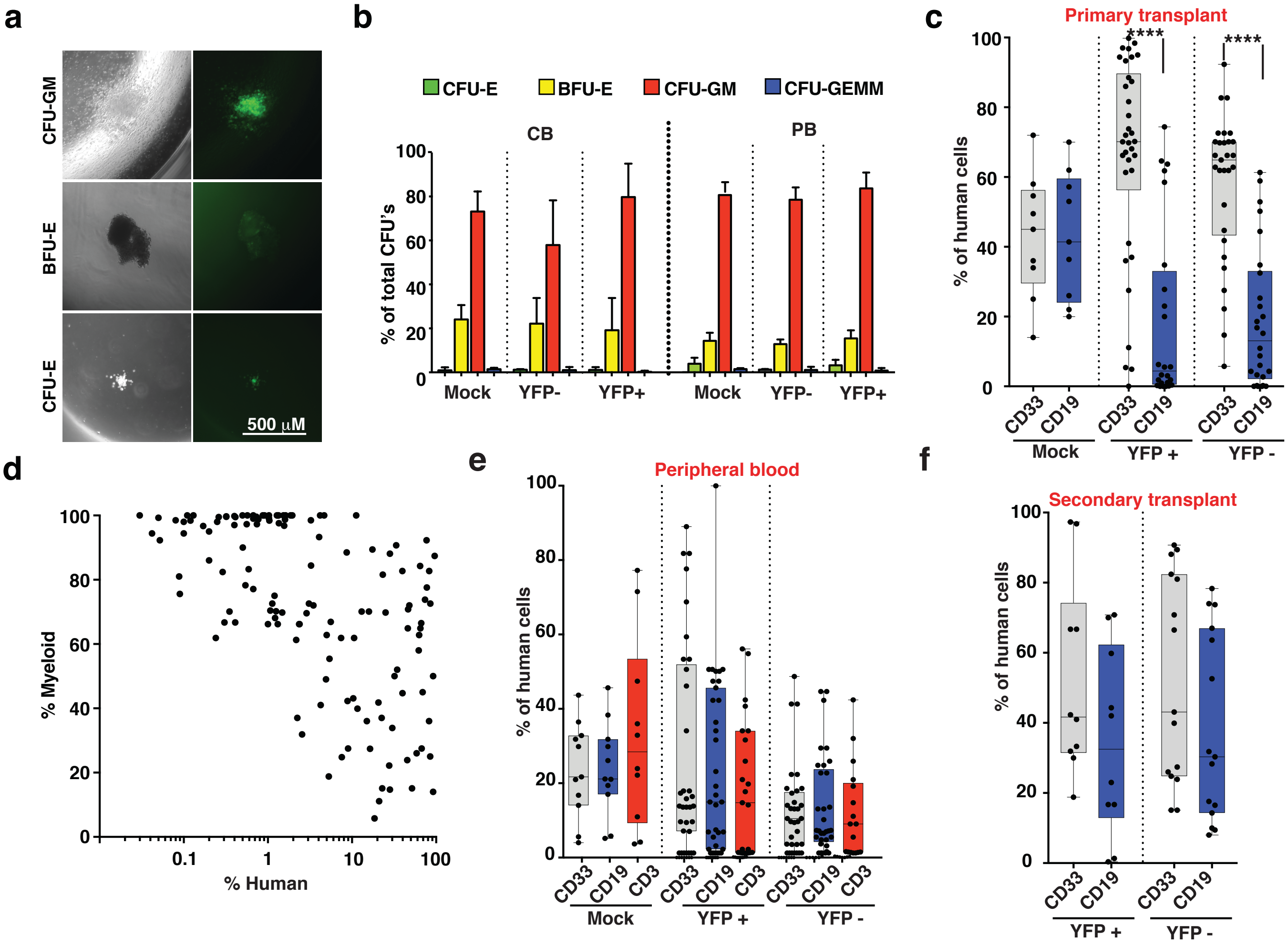

Supplementary Figure 5 | IDUA-HSPCs maintain multi-lineage differentiation potential. a, Representative photos showing morphology and YFP expression in CFU-GM, BFU-E, and CFU-E colonies. **b**, Colony formation unit frequency in mock, YFP- and YFP+ cells (only cells modified with PGK driven cassettes are shown). **c,** Box plot with whiskers (min to max) showing percent human CD33+ (myeloid), CD19+ (B) cell in the BM of mice transplanted with mock, and FAC-sorted YFP+ and YFP- cells in primary transplant mice. Each point represents data from a single mouse. **d**, XY plot showing the relationship between increasing human cell chimerism and percent of myeloid cells in the bone marrow. **e,** Percent human CD33+, CD19+, and CD3+ (T) cells in the PB of mice transplanted with mock, and FAC-sorted YFP+ and YFP- cells. **f**, Box plot with whiskers (min to max) showing percent human CD33+ (myeloid), CD19+ (B) cell in the BM secondary transplant mice. ****: $p < .0001$ in one-way ANOVA test. Post hoc comparisons were made with the Tukey's multiple comparisons test. Source data are provided as a Source Data file.

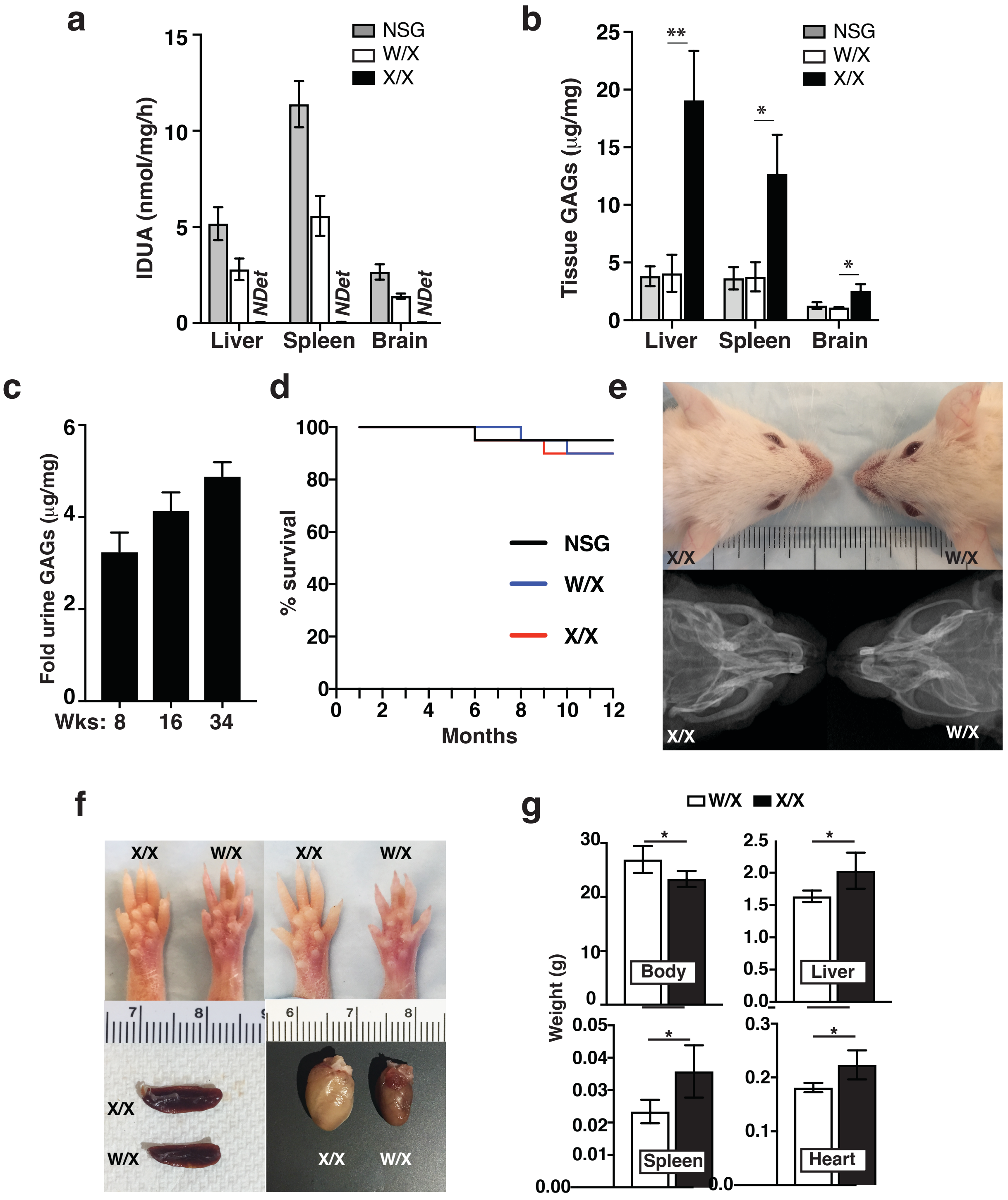

Supplementary Figure 6 | Biochemical characterization of NSG-IDUA$^{X/X}$ mice. a, Tissue IDUA enzyme activity in tissues. Ndet= Not detectable. **b,** Tissue GAGs measure by dimethylmethylene blue reactivity. **c,** Age related progression of urinary GAGs excretion. **d,** Survival analysis comparing NSG, W/X, and X/X mice during one year of observation. **e,** Representative photo of facial dysmorphism. **f,** Visceral enlargement and paw appearance. **g,** Total body, liver, spleen and heart weight in W/X and X/X mice. For **a, b, and c** data is shown as mean ± SD from 5 mice in each condition. For **d and g**, n=10 mice. Comparisons between groups were performed using unpaired t-test. *: $p < .05$, **: $p < .01$. Source data are provided as a Source Data file.

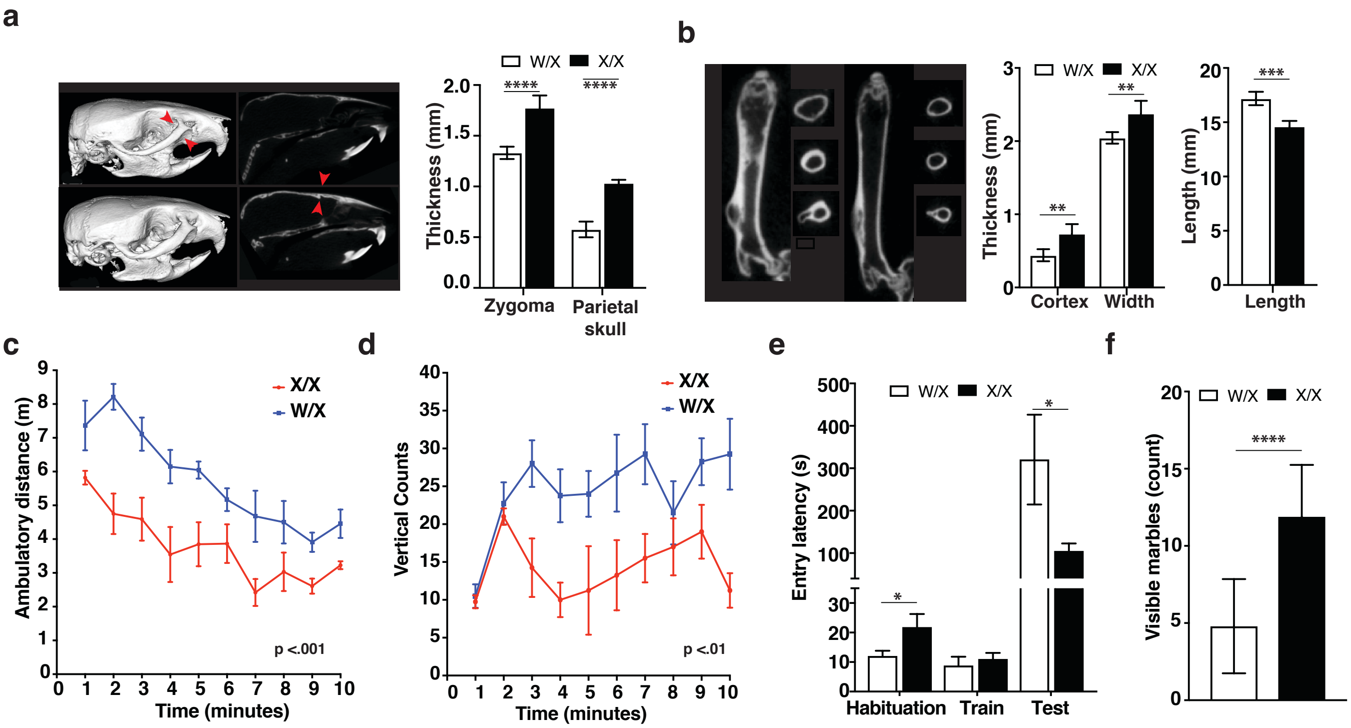

Supplementary Figure 7 | Phenotypic characterization of NSG-IDUA$^{X/X}$ mice. a, Reconstructed micro-CT images of skull and zygomatic and parietal bone thickness. **b**, CT longitudinal sections of femurs, and cortical thickness, width, and length measurements. **c**, Spontaneous locomotion in open field testing. **d,** Vertical rearing counts for W/X and X/X mice during 10-minute observation in the open field chamber. **e**, Long-term memory in passive inhibitory avoidance test. **f**, Defensive digging in the marble burying task. Total 5 female mice per genotype. Data are presented as mean ± SD for a, b, and f and mean ± SEM for c-e. Comparisons between groups were performed using unpaired t-test. *: $p < .05$, **: $p < .01$, ***: $p < .001$, and ****: $p < .0001$. Open field testing was analyzed using within-subject modeling for the entire time course by calculating the are under the curve for each mouse and comparing between genotypes with a t-test. Source data are provided as a Source Data file.

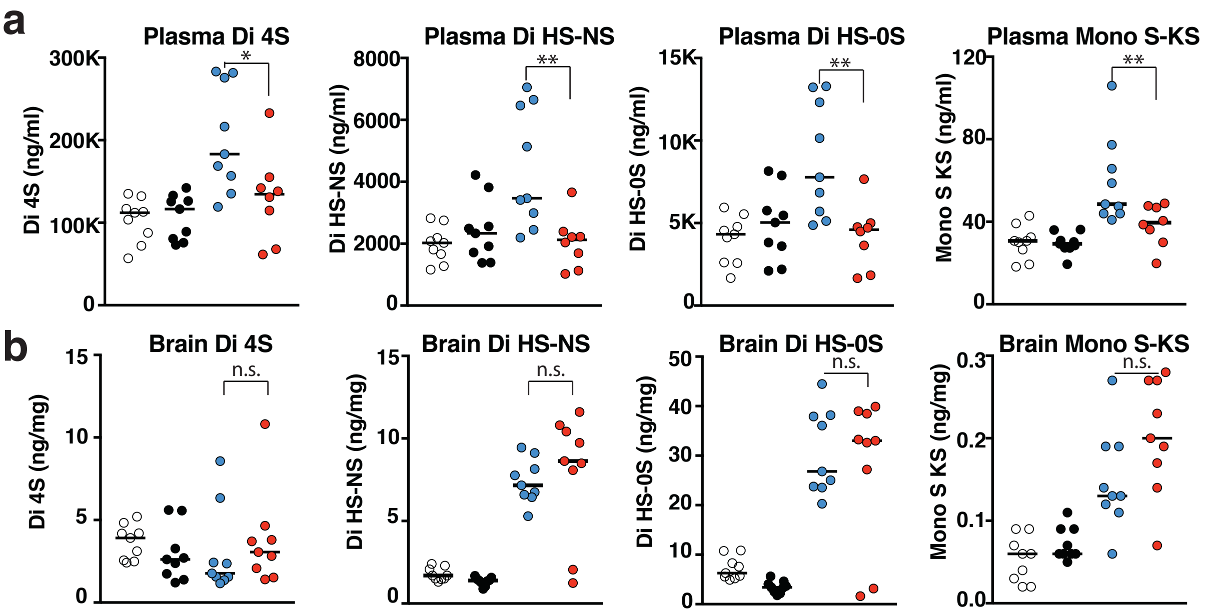

Supplementary Figure 8 | GAG quantification and disaccharide composition by LC-MS/MS. a, Plasma and **b,** Brain 18 weeks post-transplantation with bulk IDUA-HSPCs. Di 4S: Dermatan sulfate. Di HS-NS and Di HS-0S: Heparan sulfate, Mono K-KS; mono-sulfated Keratan sulfate. Each dot represents a mouse.

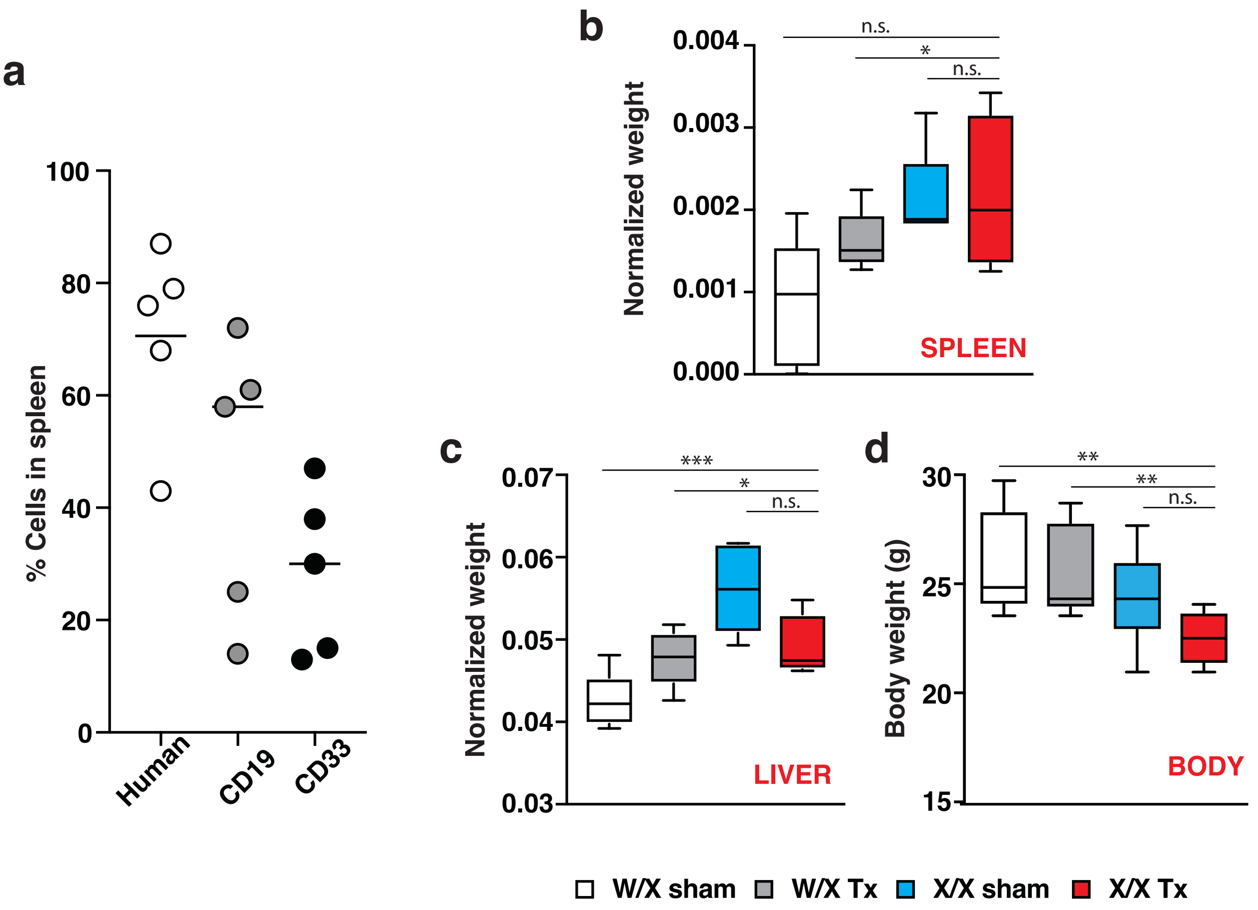

Supplementary Figure 9 | Human cell engraftment in spleen, body and organ weight after transplantation with IDUA-HSPCS. **a,** Percent human (clear), B (CD19+, gray), and myeloid (CD33+, black) cells in the spleen of X/X Tx mice 18 weeks post-transplant; Median shown. **b**, Normalized spleen weight in heterozygous transplanted (W/X Tx- dark gray), heterozygous sham-treated (W/X sham-clear), homozygous transplanted (X/X Tx- red), and homozygous sham-treated (X/X Tx- blue) mice. **c**, Normalized liver weight in the same mice. **d,** Body weigh in grams in the same mice. For **b-d** Data shown as box and whiskers (min to max). Comparisons between groups were performed using unpaired t-test. *: p < .05, **: p < .01, and ***: p < .001. Source data are provided as a Source Data file.

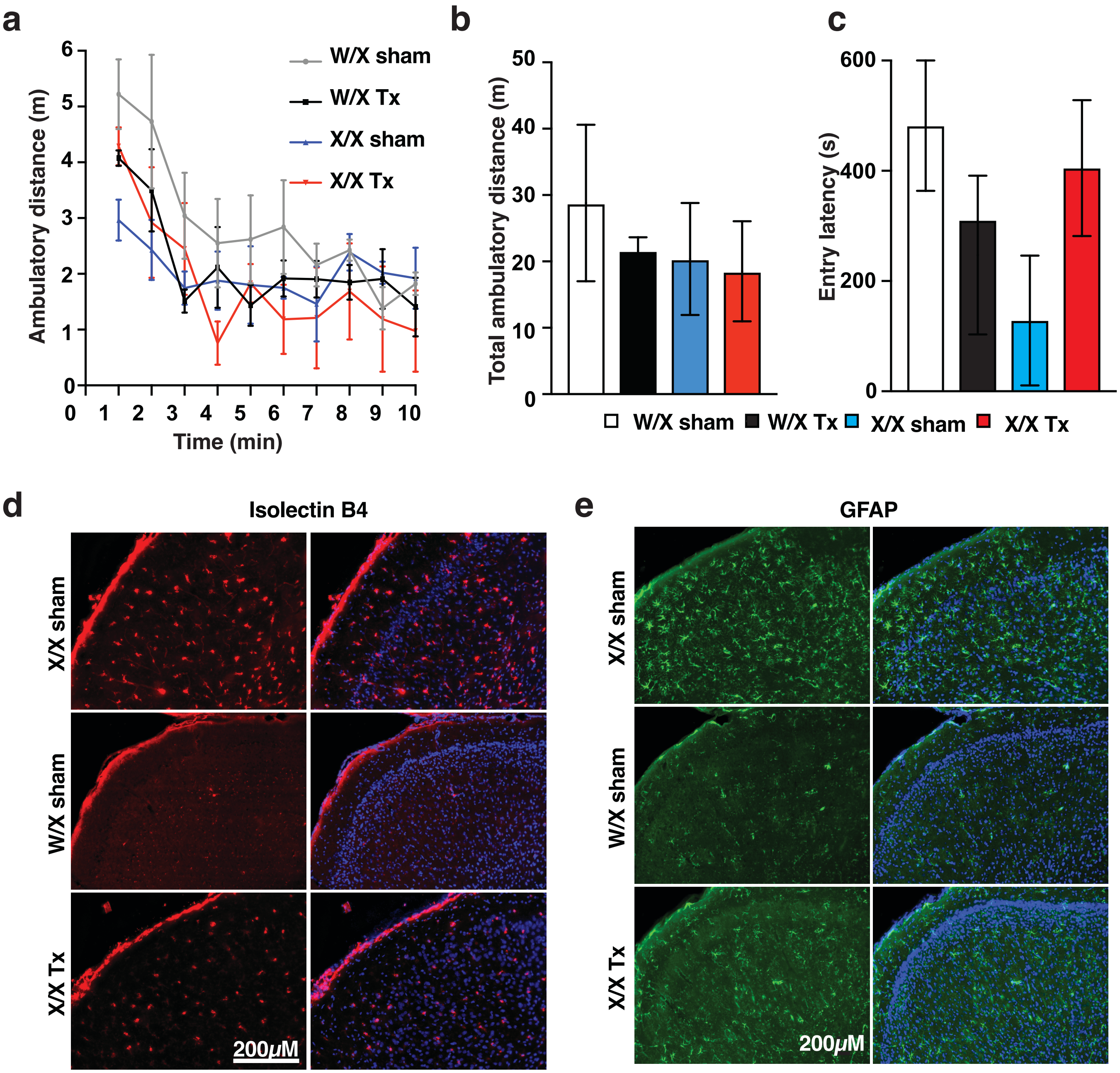

Supplementary Figure 10 | Additional studies on CNS phenotype after transplantation of IDUA-HSPCs. **a-c,** Neurobehavioral studies in mice transplanted with IDUA-HSPCs in bulk. **a,** 10-minute time course of spontaneous locomotor behavior in heterozygous transplanted (W/X Tx-black), heterozygous sham-treated (W/X sham- clear or gray), homozygous transplanted (X/X Tx- red), and homozygous sham-treated (X/X Tx- blue) mice. **b,** Total ambulatory distance in 10 minutes. **c,** Memory for inhibitory avoidance training (24h). Comparisons between groups were performed using one-way ANOVA test and post-hoc comparisons were made with the Tukey's multiple comparisons test. Open field testing and vertical rearings were analyzed using within-subject modeling by calculating the are under the curve for each mouse for the entire time course and comparing between groups with one-way ANOVA. No comparison was found to be significant. **d-e,** Immunohistochemical studies in mice transplanted with sorted IDUA-HSPCs. Representative sections of cerebral cortex showing staining for microglia (Isolectin B4-red), astrocytes (GFAP-green), and cell nuclei (Hoechst-blue).

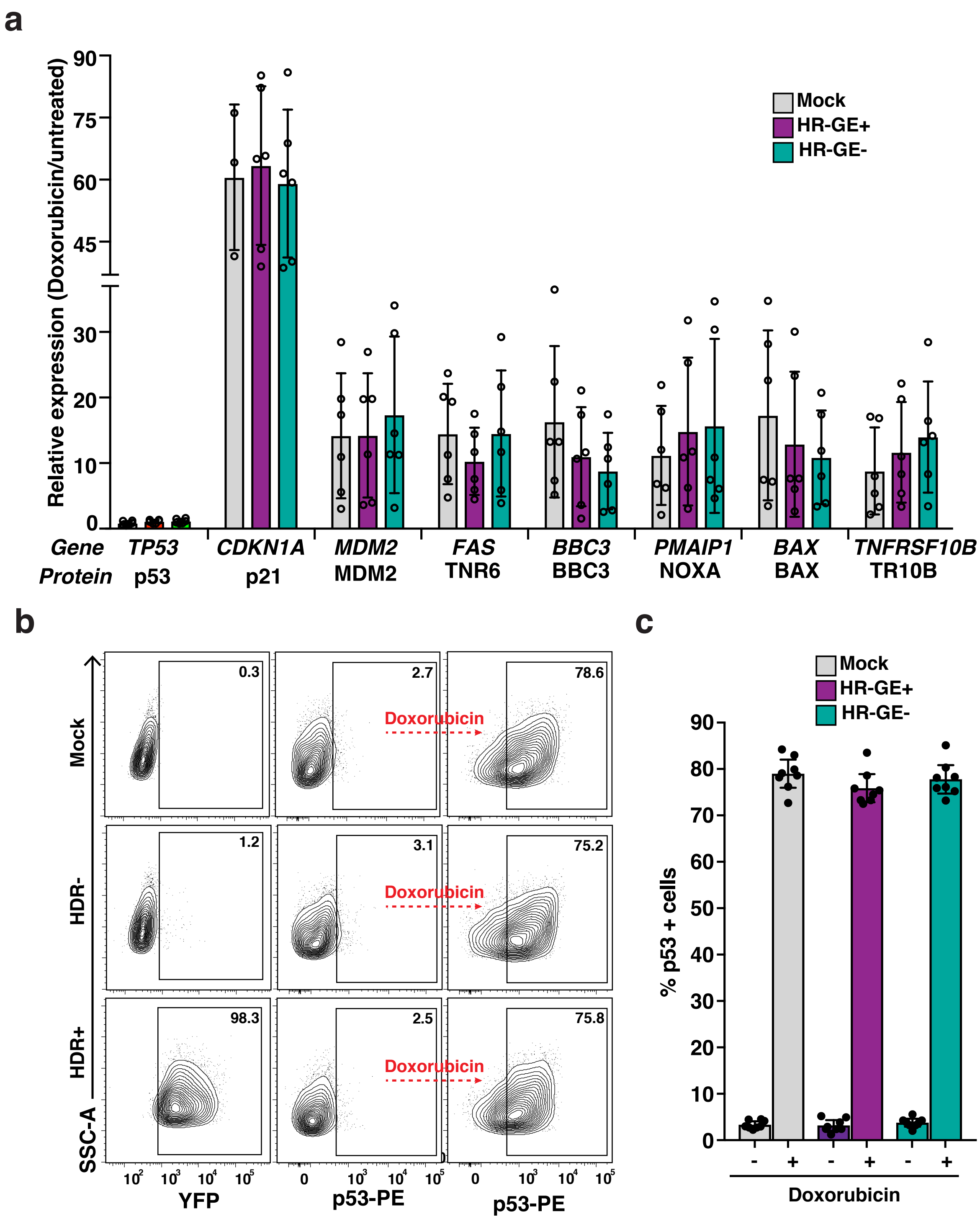

Supplementary Figure 11 | Studies on p53 function in genome edited HSPCs. a, qPCR quantification of p53 target genes represented as relative expression in doxorubicin treated compared to untreated cells. Populations of IDUA-HSPCs compared: mock (gray), HDR+ (YFP-positive, red), and HDR- (YFP-negative, green). **b,** Representative FACs plots for same cells showing p53+ and YFP+ gates. **c,** Quantification of percent p53 positive cells in untreated and doxorubicin treated IDUA-HSPCs. Three independent biological samples with two replicates each, all replicates shown. Source data are provided as a Source Data file.

Supplementary Note 1. Efficiency of modification at the CCR5 locus

To better quantify and characterize the genomic modifications at the *CCR5* loci, we measured the fraction of targeted alleles in bulk DNA preparations of cell modified with all three cassettes using droplet digital PCR (ddPCR). We first designed hydrolysis probes that amplified either the 5' or 3' homology arm with one primer inside the exogenous sequence and the second outside of the homology arm and established their accuracy with genomic DNA from a mono-allelic colony (50% allele fraction) as template (**Supplementary Figure 2c, d**). Based on this a probe that straddled the 3' homology arm was selected, and the fraction of modified alleles was obtained by using a second reference probe to the *CCRL2* gene also on chromosome. The mean fraction of modified alleles in CB-HSCPs for the SFFV and PGK driven donors was 23% ± 6, 21% ± 8 respectively, and 41% ± 5 for the cassette lacking YFP. In PB-HSPCs the modified allele fractions were 12% ± 3, 12% ± 2, and 26% ± 7 respectively (**Supplementary Figure 2e**). For a given cell preparation, knowing the fraction of alleles that were targeted and the fraction of modified cells (based on reporter expression) allowed us to calculate the distribution of cells with a one or two CCR5 alleles modified (mono-allelic vs. bi-allelic, see **Supplementary Table 1**).

Supplementary Note 2. Macrophage differentiation

We cultured mock, and targeted HSPCs in the presence of a cytokine cocktail containing stem cell factor (SCF), interleukin 3 (IL3), interleukin 6 (IL6), FMS-like tyrosine kinase 3 ligand (Flt3L), M-CSF, and GM-CSF in non-adherent tissue culture plates (For more details see materials and methods). Every 48 hours non-adherent cells were removed, transferred to a new plate and fresh differentiation media added. The adherent cells, which showed morphologic features of differentiation were maintained in the same plates in maintenance medium containing RPMI supplemented with 10% FBS and M-CSF. After three weeks adherent cells were tested for monocyte/macrophage markers by flow cytometry. Incubation in the differentiation media led to increased expression of CD11b and CD14 markers (**Fig. 2e and Supplementary Figure 3a)**. The resultant adherent cells had the typical fried egg morphology described for macrophages and exhibited robust phagocytic activity when incubated with labeled E-coli bioparticles (**Supplementary Figure 3b**). Together these data indicated that our *in vitro* differentiation protocol was effective at generating HPSC-derived macrophages.

Supplementary Note 3. A new MPSI model capable of engrafting human cells

We used CRISPR/Cas9 to knock-in a W401X mutation, analogous to the W402X mutation commonly found in patients with severe MPSI, into NSG mouse embryos as described in materials and methods. NSG-W401X homozygous mice (NSG-IDUA$^{X/X}$) replicated the phenotype of patients affected with MPSI and previously described immunocompentent[17,18] and immunocompromised[19] MPSI mice . NSG-IDUA$^{X/X}$ mice had no detectable IDUA activity in the brain, liver, and spleen (**Supplementary Figure 6a)**, and accumulated glycosaminoglycans (GAGs) as shown by increased dimethylmethylene blue reactivity in extracted tissues (**Supplementary Figure 6b**). As expected, urinary GAG excretion increased progressively with age (**Supplementary Figure 6c**). NSG-IDUA$^{X/X}$ mice exhibited the anticipated symptomatology, including progressive dysmorphism (broadening of face, flattening of the snout, and thickening of the paws), bone dysplasia, and visceral enlargement, and their lifespan was the same as NSG mice (**Supplementary Figure 6d-g**). The skeletal defect was quantitated using whole body micro–computed tomography (micro-CT). Compared to wild-type and heterozygous litter mates (NSG-IDUA$^{W/X}$), homozygous mice had thicker bones throughout. We focused on measurements of the skull and femurs where NSG-IDUA$^{X/X}$ mice had thicker zygoma and parietal skull, increased cortical thickness, and decreased femur length (**Supplementary Figure 7a, b)**.

We examined the open field behavior, passive inhibitory avoidance, and marble-burying behavior of NSG-IDUA$^{X/X}$ mice at 6 months. Compared to litter mate heterozygous controls, NSG-IDUA$^{X/X}$ mice showed reduced locomotion and decreased exploratory behavior as evidenced by a decrease in the total distance ambulated during the 10-minute interval and lower number of rearings (**Supplementary Figure 7c, d)**. NSG-IDUA$^{X/X}$ mice displayed shorter crossing times in the passive inhibitory avoidance task, and buried fewer marbles in the marble test (**Supplementary Figure 7e, f).** These defects in spontaneous locomotion, long-term memory retention, and anxiety-like behaviors were consistent with neurobehavioral abnormalities previously described in MPSI mice[20-22]. Measurement of distortion product otoacoustic emissions (DPOEs) and auditory brainstem responses (ABRs) showed that NSG mice have early-onset hearing loss rendering us unable to study this trait (data not shown).

Supplementary Table 1. Distribution of cells with mono-allelic vs. bi-allelic *CCR5* modification.

The following expression was used: *Fraction alleles modified= Fraction YFP positive x (Fraction bi-allelic + Fraction mono-allelic/2)* and where *Fraction bi-allelic + Fraction mono-allelic=1.*

	HSPC	Mean cell	Mean allele	Predicted Mono	Predicted Bi
SFFV-IDUA-	CB	34	23	0.65	0.35
PGK-IDUA-	CB	32	21	0.69	0.31
PGK-IDUA	CB	54	41	0.48	0.52
SFFV-IDUA-	PB	21	12	0.86	0.14
PGK-IDUA-	PB	24	12	1.00	0.00
PGK-IDUA	PB	44	26	0.82	0.18

Supplementary Methods

Antibodies Used

Antibody	Vendor	Catalog #	Clone	Lot ID	Dilution
Anti-human CD34-APC	Biolegend	343510	581	B234734	N/A
Anti-human CD14B-V510	Biolegend	301842	M5E2	B237626	N/A
Anti-human CD11b-PE	Biolegend	101208	M1/70	B242593	N/A
Anti-human CD45-Pacific blue	Biolegend	304029	HI30	B218608	N/A
Anti-human CD3 - Percp/Cy5.5	Biolegend	300328	hit3a	B208442	N/A
Anti-mouse mter-119 PECy5	Thermo Fisher Scientific	15592182	ter119	E062501631	N/A
Anti-mouse CD45.1-PECy7	Thermo Fisher Scientific	12045381	A20	4346378	N/A
Rabbit Anti-LAMP1	Abcam	24170	1D4B		1:200
Anti-human CD19-APC	BD Biosciences	564978	HIB19	7108612 and 6312515	N/A
Anti-human CD33-PE	BD Biosciences	561816	WM53	7157694	N/A
Anti-human CCR5-APC	BD Biosciences	556903	2D7/CCR5	5037862	N/A
Rabbit Anti-mouse GFAP	Thermo Fisher Scientific	PA316727			1:500

Analysis of glycosaminoglycans by LC/MS-MS.

Frozen brain tissues (40-60 mg) were homogenized in 1.5 ml cold acetone for 10 second using Polytron (Mount Holly, NJ) setting power 2-3. Samples were centrifuge for 30 min. at 12,000 x g at 4°C. The acetone was removed, and pellets were dried under vacuum centrifuge. The pellets were applied to 200 µl of 0.5 N NaOH and incubated at 50°C for 2 h. Samples were neutralized by adding 100 µl of 1N HCl, adjusting pH 7.0. Samples were added with sodium chloride powder (final concentration 3M) followed by centrifuge at 10,000 rpm for 5 min. at RT. The supernatants were transferred to new tubes. The tubes were added to 83.3 µl of 1N HCL to make very acidic pH (around 1.0) then tubes were centrifuge at 10,000 rpm for 5 min. at RT. The supernatants were collected to new tubes and pH was neutralized by adding 83.3 µl of 1N NaOH to make sure pH is around 7.0. The samples were added to 2 volume (935 µl) of 1.3% potassium acetate (in 100% ethanol). The samples were centrifuge at 12,000 x g for 30 min. at 4°C. The supernatants were removed, and pellets were washed with 80% cold ethanol. Finally, pellets were dried at RT and dissolved in 100 µl of 50 mM, Tris–HCl buffer (pH 7.0) and kept in -20°C until run on mass spec.

Ten microliters of each plasma/serum, urine sample or standard and 90µl of 50mM Tris–hydrochloric acid buffer (pH 7.0) was placed in wells of AcroPrep™ Advance 96-Well Filter Plates that have Ultrafiltration Omega 10 K membrane filters (PALL Corporation, NY, USA). A cocktail of 40µl with chondroitinase B (0.5mU/sample), heparitinase, and keratanase II (both 1mU/sample), and IS solution (5µg/ml) followed by 60µl of 50mM Tris-hydrochloric acid buffer was added to each well. The filter plate was placed on a receiver 96 wells plate and incubated at 37°C overnight. The plate was centrifuged at 2200 x g for 15 min. The processed samples were injected to LC-MS/MS

The chromatographic system consisted of 1260 Infinity Degasser, binary pump, autoinjector, thermostatic column compartment, and 1290 Infinity Thermostat (Agilent Technologies, Palo Alto, CA, USA) and a Hypercarb column (2.1 mm i.d. 50 mm, 5 µm, Fisher Scientific, Pittsburg, PA, USA) with hypercarb guard (2.1 mm i.d. 10 mm, 5 µm, Cole-Parmer, IL, USA) was used. The column temperature was kept at 60°C. The mobile phases were 100mM ammonia (A) and 100% acetonitrile (B). The gradient condition was programmed as follows: The initial composition of 100% A was held for 1 min, linearly modified to 30% B to 4 min, maintained at 30% B to 5.5 min, returned to 0% B to 6 min, and maintained at 0% B until 10 min. The flow rate was 0.7 ml/min. The 6460 Triple Quad mass spectrometer (Agilent Technologies) was operated in the negative ion detection mode with thermal gradient focusing electrospray ionization (Agilent Jet Stream technology, AJS). The parameters of Jet Stream technology were as below. Drying gas temperature, 350°C; drying gas flow, 11 L/min; nebulizer pressure, 58 psi; sheath gas temperature, 400°C; sheath gas flow, 11 L/min; capillary voltage, 4,000 V; and nozzle voltage, 2,000 V. Specific precursor and product ions, m/z were used to quantify each disaccharide, respectively (IS,354.3, 193.1; DS, 378.3, 175.1; mono-sulfated KS, 462, 97; di-sulfated KS, 542, 462; diHSNS,416, 138; diHS-0S, 378.3, 175.1). DS was measured as di-0S after digestion of di-4S by a 4S-sulfatase present in the preparation of chondroitinase B. The injection volume was 5µL with a total run time of 10 min per sample. The peak areas for all components were integrated automatically using QQQ Quantitative Analysis software (Agilent Technologies), and peak area ratios (area of analyses/area of IS) were plotted against concentration by weighted linear regression. Raw data of LC-MS/MS were automatically preserved. The concentration of each disaccharide was calculated using QQQ Quantitative Analysis software.

Quantitative PCR for p53 target genes. Primer list.

Gene	Forward Primer	Reverse Primer
TP53	5’-CAGTTCCTG CATGGGCGGCA-3’	5’-CGCCGGTCTCTCCCAGGACA-3’
CDKN1A (*P21*)	5’-GACCAGCATGACAGATTTC-3’	5’-TGAGACTAAGGCAGAAGATG-3’
MDM2	5’-TGGCGTGCCAAGCTTCTCTGT-3’	5’-ACCTGAGTCCGATGATTCCTGCT-3’
BBC3 (*PUMA*)	5’-ACGACCTCAACGCACAGTACGA-3’	5’-GTAAGGGCAGGAGTCCCATGATGA-3’
PMAIP1 (*NOXA*)	5’-AAGAAGGCGCGCAAGAAC-3’	5’-TCCTGAGCAGAAGAGTTTGG-3’
BAX	5’-GGCCGGGTTGTCGCCCTTTT-3’	5’-AACAGCCGCTCCCGGAGGAA-3’
TNFRSF10 B (*KILLER*)	5’-GTGTCCACAAAGAATCAGGCAT-3’	5’-CCAGGTCGTTGTGAGCTTCT-3’
FAS	5’-TGAAGGACATGGCTTAGAAGTG-3’	5’-GGTGCAAGGGTCACAGTGTT-3’
UBC	5’-GATCGCTGTGATCGTCACTT-3’	5’-TCTTTGCCTTGACATTCTCG-3’
