# Supplementary material for: Human genome-edited hematopoietic stem cells phenotypically correct Mucopolysaccharidosis type I
Source: Nat Commun. 2019 Sep 6;10:4045. doi: 10.1038/s41467-019-11962-8 (PMC6731271; doi:10.1038/s41467-019-11962-8)
Supplement: Supplementary file 2 — Reporting Summary [file 41467_2019_11962_MOESM2_ESM.pdf]

nature research

Corresponding author(s): Matthew H. Porteus, MD, PhD
Natalia Gomez-Ospina, MD, PhD

Last updated by author(s): Jul 24, 2019

Reporting Summary

Nature Research wishes to improve the reproducibility of the work that we publish. This form provides structure for consistency and transparency in reporting. For further information on Nature Research policies, see Authors & Referees and the Editorial Policy Checklist.

Statistics

For all statistical analyses, confirm that the following items are present in the figure legend, table legend, main text, or Methods section.

n/a	Confirmed	
☐	☒	The exact sample size (n) for each experimental group/condition, given as a discrete number and unit of measurement
☐	☒	A statement on whether measurements were taken from distinct samples or whether the same sample was measured repeatedly
☐	☒	The statistical test(s) used AND whether they are one- or two-sided<br>*Only common tests should be described solely by name; describe more complex techniques in the Methods section.*
☒	☐	A description of all covariates tested
☐	☒	A description of any assumptions or corrections, such as tests of normality and adjustment for multiple comparisons
☐	☒	A full description of the statistical parameters including central tendency (e.g. means) or other basic estimates (e.g. regression coefficient) AND variation (e.g. standard deviation) or associated estimates of uncertainty (e.g. confidence intervals)
☐	☒	For null hypothesis testing, the test statistic (e.g. F, t, r) with confidence intervals, effect sizes, degrees of freedom and P value noted<br>*Give P values as exact values whenever suitable.*
☒	☐	For Bayesian analysis, information on the choice of priors and Markov chain Monte Carlo settings
☒	☐	For hierarchical and complex designs, identification of the appropriate level for tests and full reporting of outcomes
☒	☐	Estimates of effect sizes (e.g. Cohen's d, Pearson's r), indicating how they were calculated

Our web collection on statistics for biologists contains articles on many of the points above.

Software and code

Policy information about availability of computer code

Data collection

1. Snapgene 4.2 (GSL Biotech LLC) was used for planning, visualizing, and documenting cloning projects
2. QuantaSoft (Bio-Rad) was used to analyze ddPCR data
3. BD FACSDIVA (Becton Dickinson) software was included in the BD FACSaria II and BD FACSCanto II analyzer and was used to collect flow cytometry data
4. SoftMax Pro 7 for data Acquisition and analysis with the SpectraMax M3 plate reader in enzymatic studies.
5. Images were acquired using NIS elements software (Nikon)
6. Ethovision (Noldus Information Technology, the Netherlands) was used for data collection in open-field testing
7. GEMINI software (San Diego Instruments) was used for data collection in passive inhibitory avoidance task
8. Prepared DNA libraries were sequenced on an Illumina MiSeq instrument. MiSeq-generated images were converted into nucleotide sequences and base quality scores in the bcl format using Illumina RTA software (v1.18.54).

Data analysis

1. Genious (Biomatters) was used for sequence analysis
2. FlowJo 10.5 (FlowJo, LLC) was used for analysis of flow cytometry data
3. Images were quantified using imageJ (open sourse)
4. Microview Software (Parallax innovations) was used for analysis of CT-scan data
5. COSMID is publically available and was used to predict off-target sites (https://crispr.bme.gatech.edu/)
6. TIDE: Tracking Indels by Decomposition (TIDE) was used for quantifying INDEL rates (https://www.deskgen.com/landing/tide.html)
7. Prism version 7 (GraphPad software) for graphing and statistical analysis
8. Excel for Mac version 14.7.7 (Microsoft) was used to analyze open-field data
9. Paired-end reads from MiSeq were filtered band merged into a longer single read from each pair with a minimum overlap of 30 nucleotides using Fast Length Adjustment of SHort reads software.
10. Alignments to reference sequences were performed using Burrows-Wheeler Aligner software for each barcode and percentage of insertions and deletions containing bases within a ±5-bp window of the predicted cut sites were quantified.
11. To quantitate InDels from Paired End sequenced (Illumina) reads we used the custom script: https://github.com/piyuranjan/

NucleaseIndelActivityScript/blob/master/indelQuantificationFromFastqPaired-1.0.1.pl

For manuscripts utilizing custom algorithms or software that are central to the research but not yet described in published literature, software must be made available to editors/reviewers. We strongly encourage code deposition in a community repository (e.g. GitHub). See the Nature Research guidelines for submitting code & software for further information.

Data

Policy information about availability of data

All manuscripts must include a data availability statement. This statement should provide the following information, where applicable:

- Accession codes, unique identifiers, or web links for publicly available datasets
- A list of figures that have associated raw data
- A description of any restrictions on data availability

The data that support the findings of this study are available on request from the corresponding author [N.GO].

Field-specific reporting

Please select the one below that is the best fit for your research. If you are not sure, read the appropriate sections before making your selection.

☒ Life sciences ☐ Behavioural & social sciences ☐ Ecological, evolutionary & environmental sciences

For a reference copy of the document with all sections, see nature.com/documents/nr-reporting-summary-flat.pdf

Life sciences study design

All studies must disclose on these points even when the disclosure is negative.

Sample size	No sample size calculations were performed. The number of animals used per transplant were determined by the number of cells successfully modified from a single human source divided by the intended dose. Sample size was increased by transplanting additional mice (different cell sources and different mouse litters). Final analysis is from a combination of these independent experiments.<br>For the phenotypic correction studies a pilot experiment using 5 mice per condition provided us an idea of the standard deviation and the magnitude of the effects.
Data exclusions	No datasets were excluded from analysis in this work.
Replication	The cells used in these experiments come from approximately 23 different human HSPC cell donors. Data is average of approximately 23 different transplantation experiments in a total of ~200 mice.<br>For the biochemical experiments of IDUA activity 3 different human cell donors were analyzed in triplicate.<br>For the off-target analysis, two human cell donors were used.
Randomization	Mice were randomly assigned to each experimental group and cage cohorts always consisted of mixed experimental conditions.
Blinding	Mice were analyzed in a blinded fashion. Mice were tagged with a code after transplantation. During analysis the investigator was blinded to experimental treatment (because the phenotypic difference between MPSI mice, investigators could not be blinded to genotype).

Reporting for specific materials, systems and methods

We require information from authors about some types of materials, experimental systems and methods used in many studies. Here, indicate whether each material, system or method listed is relevant to your study. If you are not sure if a list item applies to your research, read the appropriate section before selecting a response.

Materials & experimental systems

n/a	Involved in the study
☐	☒ Antibodies
☐	☒ Eukaryotic cell lines
☐	☐ Palaeontology
☐	☒ Animals and other organisms
☐	☐ Human research participants
☐	☐ Clinical data

Methods

n/a	Involved in the study
☐	☐ ChIP-seq
☐	☒ Flow cytometry
☐	☐ MRI-based neuroimaging

Antibodies

Antibodies used	1. Anti-human CD34-APC (biolegend, #343510, clone 581, lot B234734)<br>2. Anti-human CD14-BV510 (biolegend, #301842, clone M5E2, lot B237626)<br>3. Anti-human CD11b-PE (biolegend, #101208, clone M1/70, lot B242593))

4. Anti-human HLA-ABC APC-Cy7 (biolegend, #311402, clone W6/32, lot 224263)
5. Anti-human CD45-Pacific blue (biolegend, #304029, clone HI30, lot B218608)
6. Anti-human CD3 percp/Cy5.5 (biolegend, #300328, clone hit3a, lot B208442)
7. Anti-human CD19 APC (BD Biosciences, #564978, clone HIB19, lot 7108612 and 6312515)
8. Anti-human CD33 PE (BD Biosciences, #561816, clone WM53, lot7157694)
9. Anti-human CCR5-APC (BD Biosciences, #556903, clone 2D7/CCR5, lot 5037862)
10. Anti-mouse mter119 PE-Cy5 (ebioscience, #15-5921-82, ter-119, lot E06250-1631)
11. Anti-mouse CD45.1 PE-Cy7 (ebioscience, #12-0453-81, clone A20, lot 4346378)
12. Rabbit anti-LAMP1 (Abcam, ab24170, clone 1D4B). Dilution 1:200
13. Rabbit anti-mouse GFAP (Thermo Fisher Scientific, # PA3-16727). Dilution 1:500

Validation

All antibodies used here have been previously reported and are routinely used in flow cytometry studies. Per vendors' websites, they take quality control measures to ensure that all antibodies sold are valid and reproducible. All antibodies from Biolegend, Thermo and Abcam listed here are also listed in the Antibody Registry, which is part of the Resource Indentification Initiative (https://scicrunch.org/resources). Per BD Biosciences, the antibodies used in this study were routinely tested in flow cytometry.

The antibodies used herein for flow cytometry were validated by using positive (antigen positive cells) and negative (isotype) controls with the recommended antibody concentrations from the manufacturers.

Eukaryotic cell lines

Policy information about cell lines

Cell line source(s)

MPSI fibroblasts were obtained from Coriell Institute (G00798).

Authentication

Negative IDUA enzymatic activity performed in our lab.

Mycoplasma contamination

Cell lines not tested for Mycoplasma contamination.

Commonly misidentified lines
(See ICLAC register)

No commonly misidentified cell lines were used.

Palaeontology

Specimen provenance

Provide provenance information for specimens and describe permits that were obtained for the work (including the name of the issuing authority, the date of issue, and any identifying information).

Specimen deposition

Indicate where the specimens have been deposited to permit free access by other researchers.

Dating methods

If new dates are provided, describe how they were obtained (e.g. collection, storage, sample pretreatment and measurement), where they were obtained (i.e. lab name), the calibration program and the protocol for quality assurance OR state that no new dates are provided.

☐ Tick this box to confirm that the raw and calibrated dates are available in the paper or in Supplementary Information.

Animals and other organisms

Policy information about studies involving animals; ARRIVE guidelines recommended for reporting animal research

Laboratory animals

Species: Mus musculus
Strains:
1) NOD.Cg-PrkdcscidIL2rgtmlWjl/Sz (NSG) mice were developed at The Jackson Laboratory.
2) NSG-IDUA mice were developed in our lab using CRISPR-Cas9.
Age:
For all studies mice were transplanted at 6-8 weeks. Primary engraftment was measured 16 weeks post-transplantation, secondary transplants were analyzed after an additional 16 weeks (total 32 weeks). Biochemical and phenotypic correction was measured between 18-20 weeks post-transplant.
Sex:
1) For engraftment studies, we transplanted 161 NSG mice with roughly equal distribution of males and females.
2) NSG-IDUA mice characterization was performed in females.
3) Biochemical and phenotypic correction using bulk transplants was performed in females mice.
4) Biochemical correction using sorted cells was performed with mostly female mice.

Wild animals

Provide details on animals observed in or captured in the field; report species, sex and age where possible. Describe how animals were caught and transported and what happened to captive animals after the study (if killed, explain why and describe method; if released, say where and when) OR state that the study did not involve wild animals.

Field-collected samples

For laboratory work with field-collected samples, describe all relevant parameters such as housing, maintenance, temperature, photoperiod and end-of-experiment protocol OR state that the study did not involve samples collected from the field.

Ethics oversight	*Identify the organization(s) that approved or provided guidance on the study protocol, OR state that no ethical approval or guidance was required and explain why not.*

Note that full information on the approval of the study protocol must also be provided in the manuscript.

Human research participants

Policy information about studies involving human research participants

Population characteristics	*Describe the covariate-relevant population characteristics of the human research participants (e.g. age, gender, genotypic information, past and current diagnosis and treatment categories). If you filled out the behavioural & social sciences study design questions and have nothing to add here, write "See above."*
Recruitment	*Describe how participants were recruited. Outline any potential self-selection bias or other biases that may be present and how these are likely to impact results.*
Ethics oversight	*Identify the organization(s) that approved the study protocol.*

Note that full information on the approval of the study protocol must also be provided in the manuscript.

Clinical data

Policy information about clinical studies

All manuscripts should comply with the ICMJE guidelines for publication of clinical research and a completed CONSORT checklist must be included with all submissions.

Clinical trial registration	*Provide the trial registration number from ClinicalTrials.gov or an equivalent agency.*
Study protocol	*Note where the full trial protocol can be accessed OR if not available, explain why.*
Data collection	*Describe the settings and locales of data collection, noting the time periods of recruitment and data collection.*
Outcomes	*Describe how you pre-defined primary and secondary outcome measures and how you assessed these measures.*

ChIP-seq

Data deposition

☐ Confirm that both raw and final processed data have been deposited in a public database such as GEO.

☐ Confirm that you have deposited or provided access to graph files (e.g. BED files) for the called peaks.

Data access links<br>*May remain private before publication.*	*For "Initial submission" or "Revised version" documents, provide reviewer access links. For your "Final submission" document, provide a link to the deposited data.*
Files in database submission	*Provide a list of all files available in the database submission.*
Genome browser session<br>(e.g. UCSC)	*Provide a link to an anonymized genome browser session for "Initial submission" and "Revised version" documents only, to enable peer review. Write "no longer applicable" for "Final submission" documents.*

Methodology

Replicates	*Describe the experimental replicates, specifying number, type and replicate agreement.*
Sequencing depth	*Describe the sequencing depth for each experiment, providing the total number of reads, uniquely mapped reads, length of reads and whether they were paired- or single-end.*
Antibodies	*Describe the antibodies used for the ChIP-seq experiments; as applicable, provide supplier name, catalog number, clone name, and lot number.*
Peak calling parameters	*Specify the command line program and parameters used for read mapping and peak calling, including the ChIP, control and index files used.*
Data quality	*Describe the methods used to ensure data quality in full detail, including how many peaks are at FDR 5% and above 5-fold enrichment.*
Software	*Describe the software used to collect and analyze the ChIP-seq data. For custom code that has been deposited into a community repository, provide accession details.*

Flow Cytometry

Plots

Confirm that:

☒ The axis labels state the marker and fluorochrome used (e.g. CD4-FITC).

☒ The axis scales are clearly visible. Include numbers along axes only for bottom left plot of group (a 'group' is an analysis of identical markers).

☒ All plots are contour plots with outliers or pseudocolor plots.

☒ A numerical value for number of cells or percentage (with statistics) is provided.

Methodology

Sample preparation	*Describe the sample preparation, detailing the biological source of the cells and any tissue processing steps used.*
Instrument	*Identify the instrument used for data collection, specifying make and model number.*
Software	*Describe the software used to collect and analyze the flow cytometry data. For custom code that has been deposited into a community repository, provide accession details.*
Cell population abundance	*Describe the abundance of the relevant cell populations within post-sort fractions, providing details on the purity of the samples and how it was determined.*
Gating strategy	*Describe the gating strategy used for all relevant experiments, specifying the preliminary FSC/SSC gates of the starting cell population, indicating where boundaries between "positive" and "negative" staining cell populations are defined.*

☐ Tick this box to confirm that a figure exemplifying the gating strategy is provided in the Supplementary Information.

Magnetic resonance imaging

Experimental design

Design type	*Indicate task or resting state; event-related or block design.*
Design specifications	*Specify the number of blocks, trials or experimental units per session and/or subject, and specify the length of each trial or block (if trials are blocked) and interval between trials.*
Behavioral performance measures	*State number and/or type of variables recorded (e.g. correct button press, response time) and what statistics were used to establish that the subjects were performing the task as expected (e.g. mean, range, and/or standard deviation across subjects).*

Acquisition

Imaging type(s)	*Specify: functional, structural, diffusion, perfusion.*
Field strength	*Specify in Tesla*
Sequence & imaging parameters	*Specify the pulse sequence type (gradient echo, spin echo, etc.), imaging type (EPI, spiral, etc.), field of view, matrix size, slice thickness, orientation and TE/TR/flip angle.*
Area of acquisition	*State whether a whole brain scan was used OR define the area of acquisition, describing how the region was determined.*

Diffusion MRI ☐ Used ☐ Not used

Preprocessing

Preprocessing software	*Provide detail on software version and revision number and on specific parameters (model/functions, brain extraction, segmentation, smoothing kernel size, etc.).*
Normalization	*If data were normalized/standardized, describe the approach(es): specify linear or non-linear and define image types used for transformation OR indicate that data were not normalized and explain rationale for lack of normalization.*
Normalization template	*Describe the template used for normalization/transformation, specifying subject space or group standardized space (e.g. original Talairach, MNI305, ICBM152) OR indicate that the data were not normalized.*
Noise and artifact removal	*Describe your procedure(s) for artifact and structured noise removal, specifying motion parameters, tissue signals and physiological signals (heart rate, respiration).*
Volume censoring	*Define your software and/or method and criteria for volume censoring, and state the extent of such censoring.*

Statistical modeling & inference

Model type and settings	*Specify type (mass univariate, multivariate, RSA, predictive, etc.) and describe essential details of the model at the first and second levels (e.g. fixed, random or mixed effects; drift or auto-correlation).*
Effect(s) tested	*Define precise effect in terms of the task or stimulus conditions instead of psychological concepts and indicate whether ANOVA or factorial designs were used.*

Specify type of analysis: ☐ Whole brain ☐ ROI-based ☐ Both

Statistic type for inference<br>(See Eklund et al. 2016)	*Specify voxel-wise or cluster-wise and report all relevant parameters for cluster-wise methods.*
Correction	*Describe the type of correction and how it is obtained for multiple comparisons (e.g. FWE, FDR, permutation or Monte Carlo).*

Models & analysis

n/a	Involved in the study
☐	☐ Functional and/or effective connectivity
☐	☐ Graph analysis
☐	☐ Multivariate modeling or predictive analysis

Functional and/or effective connectivity	*Report the measures of dependence used and the model details (e.g. Pearson correlation, partial correlation, mutual information).*
Graph analysis	*Report the dependent variable and connectivity measure, specifying weighted graph or binarized graph, subject- or group-level, and the global and/or node summaries used (e.g. clustering coefficient, efficiency, etc.).*
Multivariate modeling and predictive analysis	*Specify independent variables, features extraction and dimension reduction, model, training and evaluation metrics.*
