# Supplementary material for: Human genome-edited hematopoietic stem cells phenotypically correct Mucopolysaccharidosis type I
Source: Nat Commun. 2019 Sep 6;10:4045. doi: 10.1038/s41467-019-11962-8 (PMC6731271; doi:10.1038/s41467-019-11962-8)
Supplement: Supplementary file 3 — Description of Additional Supplementary Files [file 41467_2019_11962_MOESM3_ESM.pdf]

**Title:** Supplementary Data 1

**Description:** Comprehensive off-target site analysis of the CCR5 sgRNA
